# Application of ionizing radiation as an elicitor to enhance the growth and metabolic activities in *Chlamydomonas reinhardtii*

**DOI:** 10.3389/fpls.2023.1087070

**Published:** 2023-02-20

**Authors:** Jin-Hong Kim, Shubham Kumar Dubey, Kwon Hwangbo, Byung Yeoup Chung, Seung Sik Lee, Sungbeom Lee

**Affiliations:** ^1^ Advanced Radiation Technology Institute, Korea Atomic Energy Research Institute, Jeollabuk-do, Republic of Korea; ^2^ Department of Radiation Science and Technology, University of Science and Technology, Daejeon, Republic of Korea

**Keywords:** *Chlamydomonas*, ionizing radiation, elicitor, growth stimulation, DNA damage response, reactive oxygen species, stress memory

## Abstract

*Chlamydomonas reinhardtii* is a eukaryotic, unicellular photosynthetic organism and a potential algal platform for producing biomass and recombinant proteins for industrial use. Ionizing radiation is a potent genotoxic and mutagenic agent used for algal mutation breeding that induces various DNA damage and repair responses. In this study, however, we explored the counterintuitive bioeffects of ionizing radiation, such as X- and γ-rays, and its potential as an elicitor to facilitate batch or fed-batch cultivation of *Chlamydomonas* cells. A certain dose range of X- and γ-rays was shown to stimulate the growth and metabolite production of *Chlamydomonas* cells. X- or γ-irradiation with relatively low doses below 10 Gy substantially increased chlorophyll, protein, starch, and lipid content as well as growth and photosynthetic activity in *Chlamydomonas* cells without inducing apoptotic cell death. Transcriptome analysis demonstrated the radiation-induced changes in DNA damage response (DDR) and various metabolic pathways with the dose-dependent expression of some DDR genes, such as *CrRPA30*, *CrFEN1*, *CrKU*, *CrRAD51*, *CrOASTL2*, *CrGST2*, and *CrRPA70A*. However, the overall transcriptomic changes were not causally associated with growth stimulation and/or enhanced metabolic activities. Nevertheless, the radiation-induced growth stimulation was strongly enhanced by repetitive X-irradiation and/or subsequent cultivation with an inorganic carbon source, i.e., NaHCO_3_, but was significantly inhibited by treatment of ascorbic acid, a scavenger of reactive oxygen species (ROS). The optimal dose range of X-irradiation for growth stimulation differed by genotype and radiation sensitivity. Here, we suggest that ionizing radiation within a certain dose range determined by genotype-dependent radiation sensitivity could induce growth stimulation and enhance metabolic activities, including photosynthesis, chlorophyll, protein, starch, and lipid synthesis in *Chlamydomonas* cells *via* ROS signaling. The counterintuitive benefits of a genotoxic and abiotic stress factor, i.e., ionizing radiation, in a unicellular algal organism, i.e., *Chlamydomonas*, may be explained by epigenetic stress memory or priming effects associated with ROS-mediated metabolic remodeling.

## Introduction

The eukaryotic unicellular green alga *Chlamydomonas reinhardtii* has been studied as a model organism to understand the photosynthesis, physiology, and genetics of both plants and algae (Harris, 2001; Merchant et al., 2007; Sasso et al., 2018). Due to the cumulative studies, *Chlamydomonas reinhardtii* has been most frequently proposed as a potential algal platform to produce biofuels, recombinant proteins, pharmaceuticals, and bio-products for industrial, medical, and nutritional uses (Rasala and Mayfield, 2015; Scranton et al., 2015; Yan et al., 2016; Charoonnart et al., 2019; Vishwakarma and Vavilala, 2019). In addition, facile transformation systems, low production costs, and the ability to secrete proteins are potential advantages of using *Chlamydomonas* as a single-celled recombinant protein production platform (Kumar et al., 2013; Rasala and Mayfield, 2015; Doron et al., 2016; Ma et al., 2022).

*Chlamydomonas* is commercially cultivated under batch/fed-batch/continuous and phototrophic/heterotrophic/mixotrophic growth conditions optimized for producing target metabolites such as starch, lipids, or recombinant proteins. Many studies have focused on strategies to enable the efficient cultivation of *Chlamydomonas* for various target metabolites. *Chlamydomonas*’s cell cycle and growth rate are associated with biomass yield and are determined by light intensity rather than light quality (Vítová et al., 2011; Kliphuis et al., 2012; Bialevich et al., 2022). Nutrient deficiencies, such as nitrogen, phosphorus, and sulfur deficiency, substantially alter the metabolic profiles of *Chlamydomonas* involved in the metabolism of lipids, amino acids, and external substances, causing cell growth inhibition and fatty acid accumulation in *Chlamydomonas* (Cakmak et al., 2012; Park et al., 2015; Yang et al., 2018). The photosynthetically assimilated carbon in *Chlamydomonas* is partly redirected from a pool of starch to neutral lipid synthesis (Li et al., 2010). Therefore, the relative proportions of starch and lipids can be modulated in a reverse relationship by altering the dynamic metabolic carbon flow (Sato and Toyoshima, 2021). In contrast, novel mixotrophic fed-batch cultivation, including systematic feeding of acetic acid and nutrients, is considered a commercially viable strategy applicable to high-density *Chlamydomonas* cultures, increasing biomass density, productivity, and the total amount of recombinant proteins (Fields et al., 2018). Genes related to DNA repair, signal transduction, and metabolite transport may contribute to the increased growth and biomass yield of *Chlamydomonas* cells, as suggested by a spaceflight production system using breathable plastic tissue culture bags (Zhang et al., 2020). In addition, two chemical compounds, WD10784 and WD30030, are synthetic inducers to increase lipid synthesis and storage in *Chlamydomonas* cells without restricting growth or biomass yield (Wase et al., 2019). Using an appropriate elicitor and optimizing light intensity/quality and various nutrients may be a core strategy to overcome adverse cultivation issues and to increase growth rates, biomass yield, and production of starch, lipids, or recombinant proteins, in algal platforms (Fan et al., 2014; Fan and Zheng, 2017; Li et al., 2020).

Ionizing radiation, such as X- and γ-rays, induces various DNA damage responses in plants as a potent genotoxic and mutagen (Kim et al., 2019). Therefore, γ-irradiation has been used to generate a mutant strain of *Chlamydomonas reinhardtii* with enhanced starch and lipid production (Baek et al., 2016; Koo et al., 2017b). Gamma irradiation at a dose of 80 Gy causes approximately 50% death and 20% nuclear degradation in *Chlamydomonas* cells, with the transcriptional enhancement of many conserved DNA damage response (DDR) genes such as *RAD51A*, *RPA70A*, *LIG1*, and *KU70* (Koo et al., 2017a; Jung et al., 2021). Upon exposure to γ-rays, *Chlamydomonas* cells display a dose-dependent change in chlorophyll fluorescence parameters for photosynthetic activity as well as a dose-dependent increase in the formation of reactive oxygen species (ROS) (Gomes et al., 2017). When irradiated with γ-rays of 1 kGy, another green alga, *Chlorella sorokiniana*, exhibited a substantial decrease in growth, chlorophyll content, and photosynthetic efficiency but a significant increase in the accumulation of neutral lipid triacylglycerol (Singh et al., 2022). These results could be associated with the transcriptional modulation of photosynthesis- and fatty acid biosynthesis-related genes, as well as the accumulation of ROS.

Recently, our preliminary experiments revealed that ionizing radiation below 10 Gy could have a stimulatory rather than inhibitory effect on the growth of *Chlamydomonas* cells, as previously reported in red pepper (*Capsicum annuum* L.) plants (Kim et al., 2005). This distinctive radiation dose-growth response cannot exclude the possibility that relatively low doses of ionizing radiation are still able to cause mutations in genomes and/or unknown detrimental alterations in cellular metabolism, as has been shown in animal models (Tang et al., 2017). However, when a certain dose range of toxicity or stress factors causes no critical functional damage to cellular metabolism, it contributes to the fitness and sustainable metabolic activities of cells by providing short- or long-term stress memory (priming) through chromatin remodeling and epigenetic regulation (Kim, 2021). Therefore, ionizing radiation may function as a stress memory or priming agent, as well as a damaging agent through ROS accumulation and oxidative stress responses (Gomes et al., 2017; Singh et al., 2022).

In this study, we investigated the counterintuitive dose-response of ionizing radiation such as X- and γ-rays and its potential as an elicitor to facilitate batch or fed-batch cultivation of *Chlamydomonas* cells. The dose-dependent effects of X- and γ-irradiation on *Chlamydomonas* cells were analyzed and evaluated in terms of growth and survival rates, apoptotic cell death, transcriptomic changes, photosynthetic activity, and chlorophyll/protein/starch/lipid content. A certain dose range of X- and γ-rays was shown to stimulate the growth and metabolite production of *Chlamydomonas* cells. We believe this study is the first example of applying a certain dose range of ionizing radiation as an elicitor to facilitate *Chlamydomonas* cultivation.

## Materials and methods

### Algal strain and cultural conditions

The unicellular green alga *C. reinhardtii* strain CC-125 cells (1.5 × 10^6^) were cultured in 50 mL liquid tris-acetate-phosphate (TAP) media in 250 mL flasks (Harris, 1989) by shaking at 25°C and 140 rpm under constant white light of 90–100 μmol photons m^−2^ s^−1^. In addition to the wild type (WT), two transgenic *C. reinhardtii* lines (*AtTHI-OE1* and *AtTHI-OE2*) harboring the *Arabidopsis thaliana Thionin 2.1* gene, which encodes antibacterial peptides, thionins, were used to investigate genotype-dependent radiation-sensitivity. Cells were harvested at 800 × *g*, dispensed, and subjected to X- or γ-irradiation as described below. The subsequent post-irradiation cultivations of the cells were performed in 10 mL fresh TAP media in 50 mL conical tubes, with an initial optical density of 0.05 at 750 nm for mock (or control) under the same culture conditions. The basal TAP medium was supplemented with 5 or 10 mM NaHCO_3_ to evaluate the synergistic effect of X-rays and sodium bicarbonate on cell growth.

### X- or γ-irradiation

Mid-exponential phase *C*. *reinhardtii* cells with an approximate optical density of 0.6 OD_750_ were harvested from 10 mL cultures in 50 mL conical tubes, resuspended in 1.8 mL of TAP medium, and dispensed into three 1.5 mL microcentrifuge tubes. Samples were subjected to X-irradiation of 3, 6, 12, or 18 Gy for 11.1, 22.2, 44.4, or 66.6 min at 160 kV and 1 mA, or 25, 50, or 100 Gy for 4.05, 8.1, or 16.2 min at 160 kV and 10 mA using a cabinet type X-ray machine (CP-160, Faxitron X-ray LLC, Lincolnshire, IL, USA). In addition, X-irradiation was repeated two or three times at 2-day intervals. For γ-irradiation, the mid-exponential phase *C*. *reinhardtii* cells from 50 mL cultures in 250 mL flasks were equally dispensed into two 50 mL conical tubes without concentrating and exposed to γ-rays of 3, 6, 12, 25, 50, or 100 Gy for 1 h, which were generated from a 3 kCi ^60^Co source at the Advanced Radiation Technology Institute (Jeollabuk-do, Korea). The absorbed radiation dose for each sample was determined using a 5 mm-diameter alanine dosimeter (Bruker Instruments, Rheinstetten, Germany), as described previously (Song et al., 2016).

### Measurement of cell growth and survival rate

Cell density was used to represent the relative growth of the mock (or control) and irradiated cells. The cell densities of *C*. *reinhardtii* cultures in liquid TAP media were obtained by measuring the OD_750_ at 2 days or consecutively for 5 days after each X- or γ-irradiation. For the survival rate, the mock and X-irradiated cells (1 × 10^3^–1 × 10^4^ cells) were spread on solid TAP medium plates and incubated at 25°C for 7 days under constant white light of approximately 90–100 μmol photons m^−2^ s^−1^. After incubation, visible colonies were counted and used to calculate the relative survival rates of mock and irradiated cells.

### Transcriptome analysis by RNA-seq

For RNA isolation, the harvested and frozen cells (1 × 10^8^) were resuspended in 1 mL of TRIzol reagent (Invitrogen, CA, United States), vortexed for 10 min, incubated for 5 min at 25°C, and centrifuged for 10 min at 4°C and 12,000 × *g*. The supernatant was mixed with 250 µL of chloroform (Sigma-Aldrich, MO, United States) by vortexing for 2 min, combined with an equal volume of phenol-chloroform (1:1 v/v; Sigma-Aldrich, MO, United States) after centrifugation, mixed by vortexing for 2 min, and centrifuged again. It was finally mixed with an equal volume of isopropanol, incubated for 45 min at 4°C, and centrifuged for 20 min at 4°C and 12,000 × *g*. The resultant RNA pellet was washed twice with 800 µL 75%(v/v) ethanol, dissolved in DEPC-treated water, and used for RNA-Seq and quantitative RT-PCR analyses.

For RNA-Seq, three biological replicates from different batches were prepared for transcriptome analysis. Total RNA was quantified using the Invitrogen Quant-IT™ RiboGreen™ RNA Assay Kit, and its integrity was assessed on the Agilent TapeStation system (Agilent Technologies, Santa Clara, CA, USA) using the RNA ScreenTape. RNA-Seq paired-end libraries were prepared using the Illumina TruSeq Stranded Total RNA Library Prep Plant Kit (Illumina, San Diego, CA, USA). The libraries were quantified using the KAPA Library Quantification Kit (Kapa Biosystems, Wilmington, MA, USA) and qualified using the Agilent TapeStation system with the D1000 ScreenTape. Sequencing of paired-end libraries was performed using the Illumina NovaSeq 6000 System (Illumina, Inc., San Diego, CA, USA).

The quality of the raw reads (FASTQ) was evaluated using the FastQC v0.11.5, and ‘dirty’ reads were removed using the Trimmomatic v0.36 to decrease data noise before downstream analysis. Briefly, the reads were subjected to the standard quality control (QC) criteria for trimming and cleaning as follows: (1) reads that aligned to primers and/or adaptors, (2) reads with over 50% low-quality bases (quality value ≤ 5) in one read, and (3) reads with over 10% unknown bases (N bases). After filtering, the remaining ‘clean’ reads were stored in FASTQ format. All high-quality clean reads were then mapped to the NCBI *Chlamydomonas reinhardtii* v5.5 or the Phytozome *Chlamydomonas reinhardtii* v6.1 reference genome using HISAT2 v2.2.1 (Kim et al., 2015a) to generate a BAM file. The BAM files were sorted and indexed with Samtools, and the number of reads matching each gene in the *Chlamydomonas reinhardtii* genome was counted using HTSeq-count v0.11.3.

Differentially expressed genes (DEGs) were identified using the read count data. Briefly, the read count data were filtered [read count < 1 (3 of 3)], and the count per million (CPM) was normalized using EdgeR software. Finally, the read count of each gene in every sample was normalized with that of the mock sample to calculate the fold change in gene expression between mock and test samples. Log_2_-transformed fold-change values > 1 were considered differentially expressed between the mock and test samples. Functional enrichment analysis of DEGs for Gene Ontology (GO) and KEGG pathway were conducted using the Gene Ontology Database and DAVID, respectively (Huang et al., 2009). The DEGs for this analysis were obtained using the Phytozome *Chlamydomonas reinhardtii* v6.1 reference genome.

### Gene expression analysis by quantitative reverse transcription-PCR

For quantitative RT-PCR, RNA isolation was performed as described in RNA-Seq analysis. cDNA was synthesized from 1 μg of each RNA sample using oligo(dT) primers and the LaboPass™ cDNA Synthesis Kit (Cosmo Genetech, Seoul, Korea). Subsequent quantitative PCR (qPCR) amplification cycle conditions were 95°C for 30 s, followed by 40 cycles of 95°C for 10 s, 57°C for 10 s, and 72°C for 1 min using the CFX Connect™ Real-Time PCR Detection System (Bio-Rad Laboratories, Hercules, CA, USA) with the iTaq Universal SYBR^®^ Green Supermix (Bio-Rad Laboratories) and gene-specific primers (**Supplementary Table 1**). The relative expression level of each gene was calculated between mock and X- or γ-irradiated samples using the comparative C_T_ method (Liu and Saint, 2002). The expression data of the three biological replicates were normalized to that of the endogenous reference gene *CrTUBA1*.

### Apoptotic cell death assay by staining with Annexin V and 7-Aminoactinomycin D

The quantitative analysis of apoptotic and necrotic dead cells was conducted using the flow cytometer Muse ™ Cell Analyzer (Merck Millipore, Billerica, MA, USA) and the Muse™ Annexin V and Dead Cell Assay Kit (MCH100105; Merck Millipore) according to the manufacturer’s instructions. The mock and irradiated *C*. *reinhardtii* cells were harvested at 6, 24, and 48 h after X-irradiation, washed twice with Dulbecco’s PBS, stained with Muse™ Annexin V and Dead Cell Reagent at a final concentration of 5 × 10^6^ cells mL ^−1^, and finally subjected to the apoptotic cell death assay using 5,000 cells for each sample. Apoptotic cell death was expressed as the proportion of living, early/late apoptotic, and dead cells, which were determined using Muse™ Cell Analyzer software (Muse 1.1.2; Merck Millipore).

### Photosynthesis assay

Chlorophyll fluorescence in *C*. *reinhardtii* cells was measured and analyzed in a 96-well plate using the IMAGING-PAM chlorophyll fluorometer (WALZ, Effeltrich, Germany) as described previously (Kim et al., 2009) to evaluate photosynthetic activity. The cells (1 × 10^8^) were harvested 48 h after the third repetitive X-irradiation, and chlorophyll fluorescence was measured. Readings were taken from the wells after a 96-well black plate containing cell cultures was dark-incubated for 15 min at 25°C. Chlorophyll fluorescence parameters Fv/Fm, ETR, qP, and NPQ were calculated as described by Kim et al. (2005) and were used as representative parameters for photosynthetic activity. The maximum electron transport rate, ETRmax, was obtained from the relative ETR *vs*. PPFD curve, as reported previously (Bischof et al., 2000).

### Determination of chlorophyll and protein content

Chlorophyll and protein contents were determined using the methods described by Kim et al. (2009) with modifications for *C*. *reinhardtii* cells. Cells (1 × 10^8^) were harvested 48 h after the second repetitive X-irradiation. The cells were subjected to vigorous vortexing in 500 μL of 100% (v/v) ice-cold acetone for chlorophyll extraction, and cell debris was removed by centrifugation at 4°C and 21,000 × *g* for 5 min. Chlorophyll concentration was calculated using the equations of Lichtenthaler (1987) as follows: chlorophyll *a* = 11.24 × A_661.6_ − 2.04 × A_644.8_; chlorophyll *b* = 21.13 × A_644.8_ − 4.19 × A_661.6_; and total chlorophyll = 18.09 × A_644.8_ + 7.05 × A_661.6_.

For protein extraction, the harvested cells (1 × 10^8^) were resuspended and shaken in 300 μL of lysis buffer containing 60 mM DTT, 60 mM Na_2_CO_3_, 2% (w/v) SDS, and 12% (w/v) sucrose for 20 min at 25°C (Ferrante et al., 2011). The protein extracts were obtained by centrifugation at 4°C and 10,000 × *g* for 1 min and mixed with five volumes of 100% (v/v) ice-cold acetone. The mixture was kept at −20°C for 2 h to facilitate protein precipitation and centrifuged at 4°C and 5,000 × *g* for 15 min. The protein pellets were washed twice with 100% (v/v) ice-cold acetone and kept in a fume hood for the complete drying of acetone. The dried protein pellets were dissolved in distilled water and used to determine the protein concentration according to the manual of the Bio-Rad Protein Assay Kit (Bio-Rad Laboratories, Hercules, CA, USA), using the method of Bradford (1976).

### Determination of starch and lipid content

Starch and lipid contents were determined using the methods of Xiao et al. (2006) and Tran et al. (2019) with some modifications. Mock and X-irradiated cells were harvested 48 h after the second repetitive X-irradiation. All experiments were performed in triplicate. The cells were incubated with 100 µL of iodine reagent (5 mM I_2_ and 5 mM KI) at a ratio of 1:1 (v/v) in a 96-well plate to determine starch content. Following color development, the absorbance of the iodine-treated samples was measured at 580 nm. The relative starch content of the mock and X-irradiated samples was calculated from the absorbance before and after normalization against OD_750_.

Neutral lipid content was quantified using a fluorescence-based microplate assay after staining mock and X-irradiated cells with Nile Red. The cells were incubated with 100 µL Nile Red (a stock solution of 2 µg mL^-1^) at a ratio of 1:1 (v/v) in a 96-well plate for 30 min in the dark at 25°C. Nile Red fluorescence signals were measured at an excitation wavelength of 560 nm and an emission wavelength of 635 nm. The relative lipid content of the mock and X-irradiated samples was calculated from the fluorescence before and after normalization against OD_750_, respectively.

### Production and scavenging assay of ROS

Production and scavenging of ROS in *C*. *reinhardtii* cells after X-irradiation with 6 Gy was estimated by measuring the cellular H_2_O_2_ content in absence and presence of ascorbic acid (AA) as a ROS scavenger. The cells (6 × 10^7^) were harvested at 1 h post-irradiation without or with 1 mM AA treatment for 1 h at 25°C, and were incubated with 10 μM 2’,7’-dichlorofluorescin diacetate (H_2_DCF-DA) for 25 min in darkness at 25°C. The cell-permeable fluorometric probe H_2_DCFDA is cleaved to 2’,7’-dichlorofluorescin (H_2_DCF) by endogenous esterases in cells and is oxidized to its fluorescent form DCF upon reaction with H_2_O_2_. DCF fluorescence signals were measured at an excitation wavelength of 485 nm and an emission wavelength of 530 nm. The ROS level of the mock and X-irradiated samples was calculated relative to the mock without AA treatment by the DCF signal.

### Statistical analysis

All experiments were repeated more than three times using biological replicates harvested after independent X- or γ-irradiation. The data were subjected to a two-sample independent *t*-test, one-way or multivariate analysis of variance followed by Tukey’s honest significance difference test, correlation or discriminant analysis using the statistical and graphical functions of R version 4.2.1 (R Core Team, 2022) and ggplot2 (Villanueva and Chen, 2019) in RStudio 2022.07.1 + 554 (Rstudio Team, 2022). A *p*-value less than 0.05 was considered significant.

## Results

### Counterintuitive growth response of *C. reinhardtii* cells after X- or γ-irradiation at different doses

**Figure 1:**
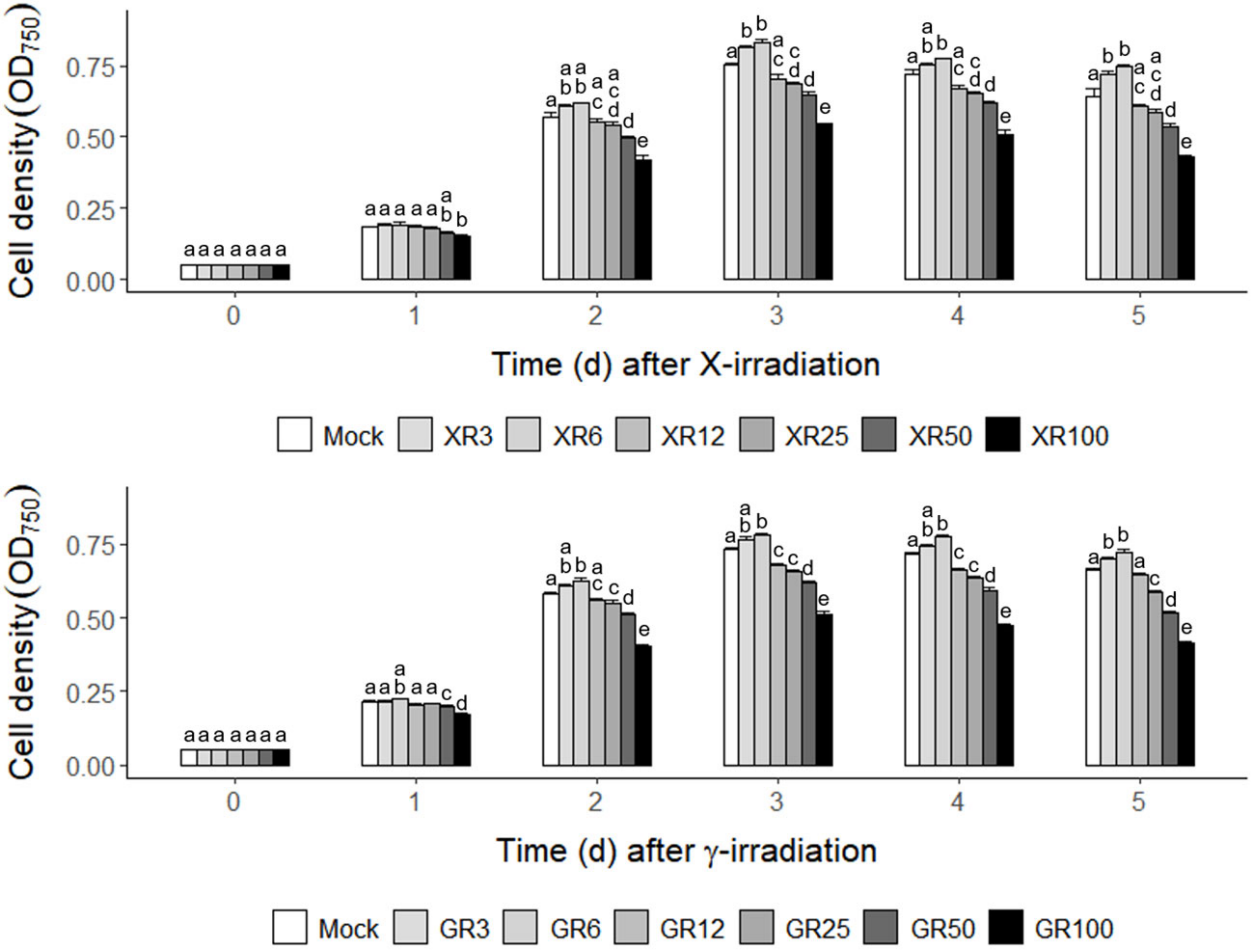
Counterintuitive dose-responses of X- or γ-irradiation in the cultivation of *C. reinhardtii* cells. **(A)** X-irradiation; **(B)** γ-irradiation. XR3 (GR3), XR6 (GR6), XR12 (GR12), XR25 (GR25), XR50 (GR50), and XR100 (GR100) represent X-rays (γ-rays) of 3, 6, 12, 25, 50, and 100 Gy, respectively. Mid-exponential phase *C. reinhardtii* cells were differentially subjected to X- or γ-irradiation to have the absorbed doses of 3, 6, 12, 50, and 100 Gy as described in the Materials and Methods section. The cell densities were compared by measuring the optical density at 750 nm (OD_750_) to evaluate the growth rates of mock and irradiated cells. Data represent the mean ± standard error (SE) with *n* = 9 from three independent experiments. Different letters indicate significant differences at *p* < 0.05 (one-way analysis of variance followed by Tukey’s honestly significant difference test).

The growth rates of *C. reinhardtii* cells were analyzed after X- or γ-irradiation with different doses of 3, 6, 12, 60, and 100 Gy to investigate the dose effect of ionizing radiation on algal cells. The two types of irradiation with the same absorbed doses but different dose rates, as described in the Materials and Methods section, revealed similar stimulatory and inhibitory effects on the growth curves of *C. reinhardtii* cells (**Supplementary Figures 1, 2**). The similar dose-response curves following the two types of irradiation demonstrated that the growth response of *C. reinhardtii* cells is dependent on the absorbed dose rather than on the dose rate. In both X- and γ-irradiation, doses of 12 to 100 Gy inhibited cell growth in a dose-dependent manner, while doses of 3 and 6 Gy increased cell growth from 2 days after irradiation (**Figure 1**). These results suggest that X- or γ-irradiation at a relatively low dose (< 10 Gy) can stimulate growth in *C reinhardtii* cells.

### Confirmation of growth stimulation of *C. reinhardtii* cells after repetitive X-irradiation with two relatively low doses, 3 and 6 Gy

**Figure 2:**
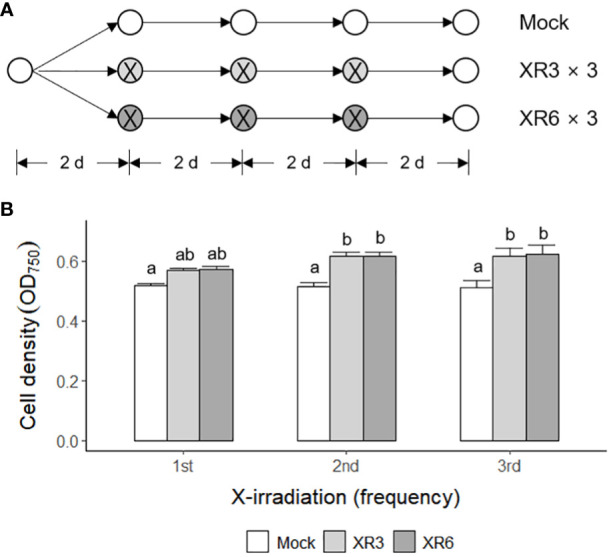
Enhanced growth stimulation of *C. reinhardtii* cells by repetitive X-irradiation with 3 or 6 Gy. XR3 and XR6 represent X-rays of 3 and 6 Gy, respectively. **(A)** A schematic diagram of repetitive X-irradiation. The absorbed doses of 3 and 6 Gy, which increased the growth of *C. reinhardtii* cells, were repeatedly applied to the X-irradiated cells at 2-day intervals as described in the Materials and Methods section. The cultivation of the mock was initiated at a cell density of 0.05 OD_750_. The irradiated samples were subjected to post-irradiation cultivation with the equivalent volume of the mock to cumulate a difference in the growth rate after each X-irradiation. **(B)** Cell density (OD_750_) of *C. reinhardtii* 2 days after single or repetitive X-irradiation. Data represent the mean ± standard error (SE) with *n* = 9 from three independent experiments. Different letters indicate significant differences at *p* < 0.05 (one-way analysis of variance followed by Tukey’s honestly significant difference test).

Subsequently, *C. reinhardtii* cells were subjected to repetitive X-irradiation at 3 or 6 Gy to substantiate the growth stimulatory effect of X- or γ-irradiation at a relatively low dose. We investigated whether repetitive X-irradiation with 3 or 6 Gy, with a final cumulative dose of 9 or 18 Gy, respectively, could further increase the growth of *C. reinhardtii* cells beyond the single X-irradiation (**Figure 2A**). The irradiation groups demonstrated 9.8−10.4% higher cell densities after the first X-irradiation, 19.2−19.6% after the second, and 20.5−21.7% after the third compared to the mock group, suggesting increasing growth stimulation at least up to the third (**Figure 2B**). In particular, repetitive X-irradiation with 6 Gy with a final cumulative dose of 18 Gy enhanced growth stimulation approximately two-fold, despite growth inhibition by single X- or γ-irradiation with doses above 12 Gy (**Figures 1**, **2**). These results suggest that repetitive X-irradiation with a certain range of relatively low doses can enhance the growth stimulation of *C. reinhardtii* beyond single X-irradiation by preventing the growth inhibition of a high cumulative dose. No substantial difference in the radiation-induced growth stimulation between the 3 and 6 Gy groups after repetitive X-irradiation may imply that the two doses are within similar equilibrium between radiation-induced positive and negative effects.

### Difference in transcriptomic profiles and expression of DDR genes between mock and X- or γ-irradiated *C. reinhardtii* cells

**Figure 3:**
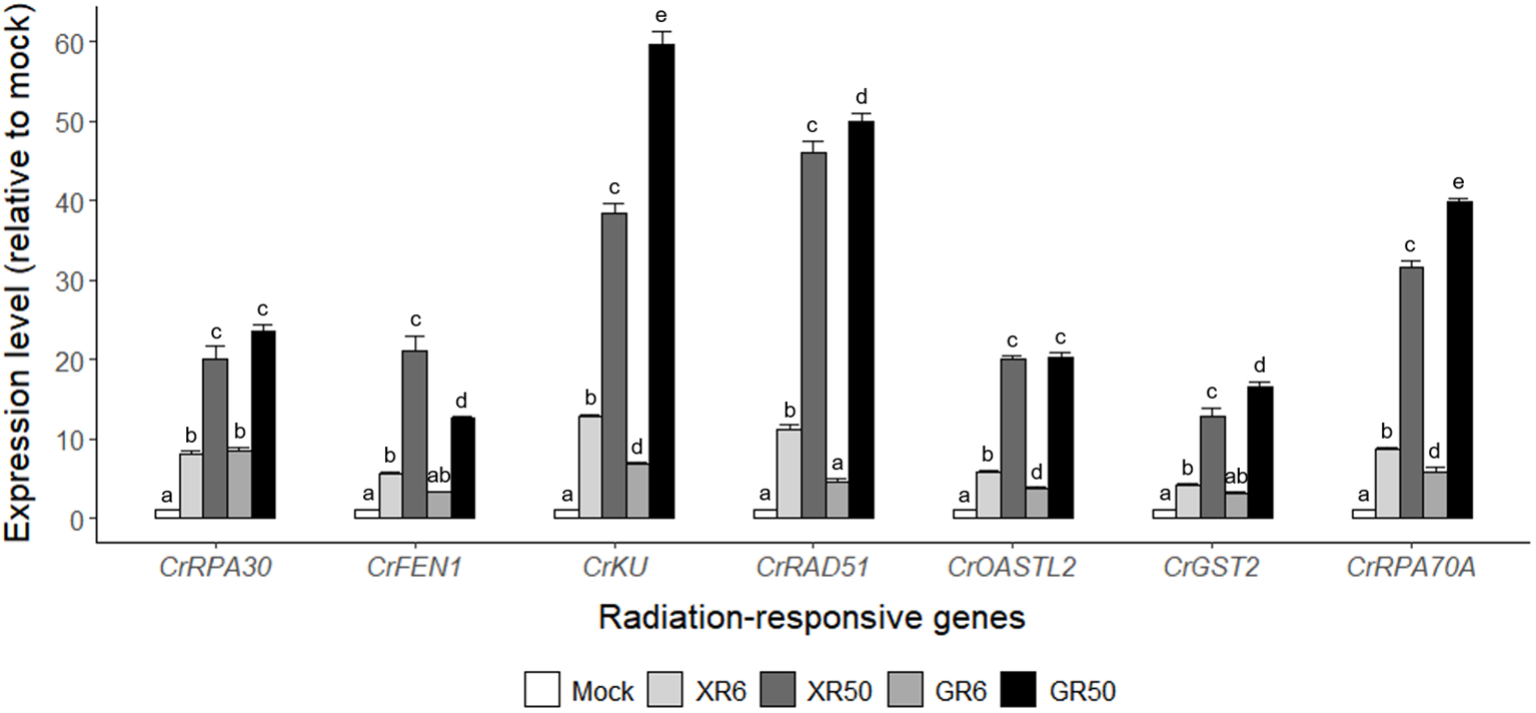
Relative transcription levels of the seven DNA damage response (DDR) genes from RNA-Seq analysis of X- or γ-irradiated *C. reinhardtii* cells. XR6 (GR6) and XR50 (GR50) represent X-rays (γ-rays) of 6 and 50 Gy, respectively. All transcription levels were measured by quantitative real-time polymerase reaction (RT-qPCR) and are shown relative to the mock by using *CrTUBA1* as an endogenous reference gene. Data represent the mean ± standard error (SE) with *n* = 9 from three independent experiments. Different letters indicate significant differences at *p* < 0.05 (one-way analysis of variance followed by Tukey’s honestly significant difference test).

Gamma irradiation at doses of 3–48 Gy induced the expression of DDR genes in a dose-dependent manner, following nuclear DNA damage in *Arabidopsis* and rice plants (Ryu et al., 2018; Kim et al., 2021). However, γ-H2AX induction and DDR gene expression were not statistically different within the dose range of 3–12 Gy and almost recovered to the control levels within 24 h. To evaluate the genotoxic stress of X- or γ-irradiation at two different doses proven to stimulate or inhibit cell growth, we compared the genome-wide transcriptomic profiles of *C. reinhardtii* by RNA-Seq analysis after X- or γ-irradiation at doses of 6 and 50 Gy. The overall transcriptome changes were discriminated by the radiation dose rather than the radiation type and manifested mainly by DDR genes (**Supplementary Figure 3** and **Table 1**). Transcription levels of the seven DDR genes selected from **Table 1** were found to increase in a dose-dependent manner but were rarely affected by the radiation type in RT-qPCR analysis (**Figure 3**). The concerted transcriptional activation of these DDR genes suggests that various types of DNA damage might be induced after X- or γ-irradiation, even at a dose of 6 Gy. Radiation-induced genotoxic stress needs to be further evaluated by physiological tests such as cell death and survival rate assays to explain the growth-stimulatory effect of X- or γ-irradiation at doses below 10 Gy.

**Table 1:** Transcriptomic profiles of *C. reinhardtii* cells after X- or γ-irradiation with two different doses.

ORF name	XR6	XR50	GR6	GR50	Gene ID	Gene name	Description
*CHLRE_02g082000v5*	*3.33*	*5.54*	*4.36*	*3.62*	*5727320*	*CrADG*	*DNA repair glycosylase*
CHLRE_02g100000v5	2.36	4.04	2.57	4.32	5725571	CrRPA30	replication protein A 30 kDa subunit
CHLRE_03g145687v5	1.85	3.4	1.29	3.25	5721250	CrFEN1	nuclease, Rad2 family
*CHLRE_04g214800v5*	*-1.71*	*N.D.*	*N.D.*	*-1.74*	*5724268*	*CrVMPL1*	*R-SNARE protein, VAMP-like family*
CHLRE_06g263050v5	1.4	2.5	1.09	3.18	5722002	CrEFP2	mitochondrial elongation factor P
*CHLRE_06g275750v5*	*N.D.*	*-1.09*	*-2.09*	*N.D.*	*5721748*	*CrHTR2*	*histone H3*
CHLRE_10g423800v5	3.16	4.3	2.96	4.82	5728221	CrKU	DNA binding protein
CHLRE_12g505500v5	2.2	2.91	1.5	1.65	5716467	CrHTR2	histone H3
CHLRE_12g509400v5	1.48	1.97	1.43	2.67	5716542	CrRIR2B	ribonucleoside-diphosphate reductase small subunit
CHLRE_14g622850v5	4.06	5.83	3.58	6.38	5718221	CrRAD51	DNA recombination protein
*CHLRE_14g632799v5*	*1.87*	*2.5*	*1.91*	*3.17*	*5718230*	*CrZSP2*	*lectin-like component of the zygote cell wall*
CHLRE_16g664250v5	2.17	3.49	1.78	3.62	5726998	CrOASTL2	cysteine synthase
CHLRE_16g682725v5	1.23	2.84	1.54	3.46	5724816	CrGSTS2	glutathione S-transferase
CHLRE_17g718850v5	3.77	4.9	3.25	5.85	5729312	CrRPA70A	replication protein A subunit
CHLRE_17g740950v5	-1.33	-1.22	-1.15	-1.34	5726658	CrLHL4	high intensity light-inducible lhc-like gene

Genes with more than a two-fold transcription change in both X- and γ-irradiated groups and a descriptive annotation for gene name were selected. XR6 (GR6) and XR50 (GR50) represent X-rays (γ-rays) of 6 and 50 Gy, respectively. Digits are log_2_-transformed of normalized fold change values. Open reading frame (ORF) names are used to alphabetically list the gene names that are temporarily attributed to an ORF by a sequencing project. Underlined genes were shown to be explicitly associated with DNA damage response (DDR) or dose-dependently induced by all the irradiations. The genes in italic have average read counts of no more than 5. N.D., not detected.

### Difference in cell death and survival rates of *C. reinhardtii* after X-irradiation with two different doses, 6 and 50 Gy

**Figure 4:**
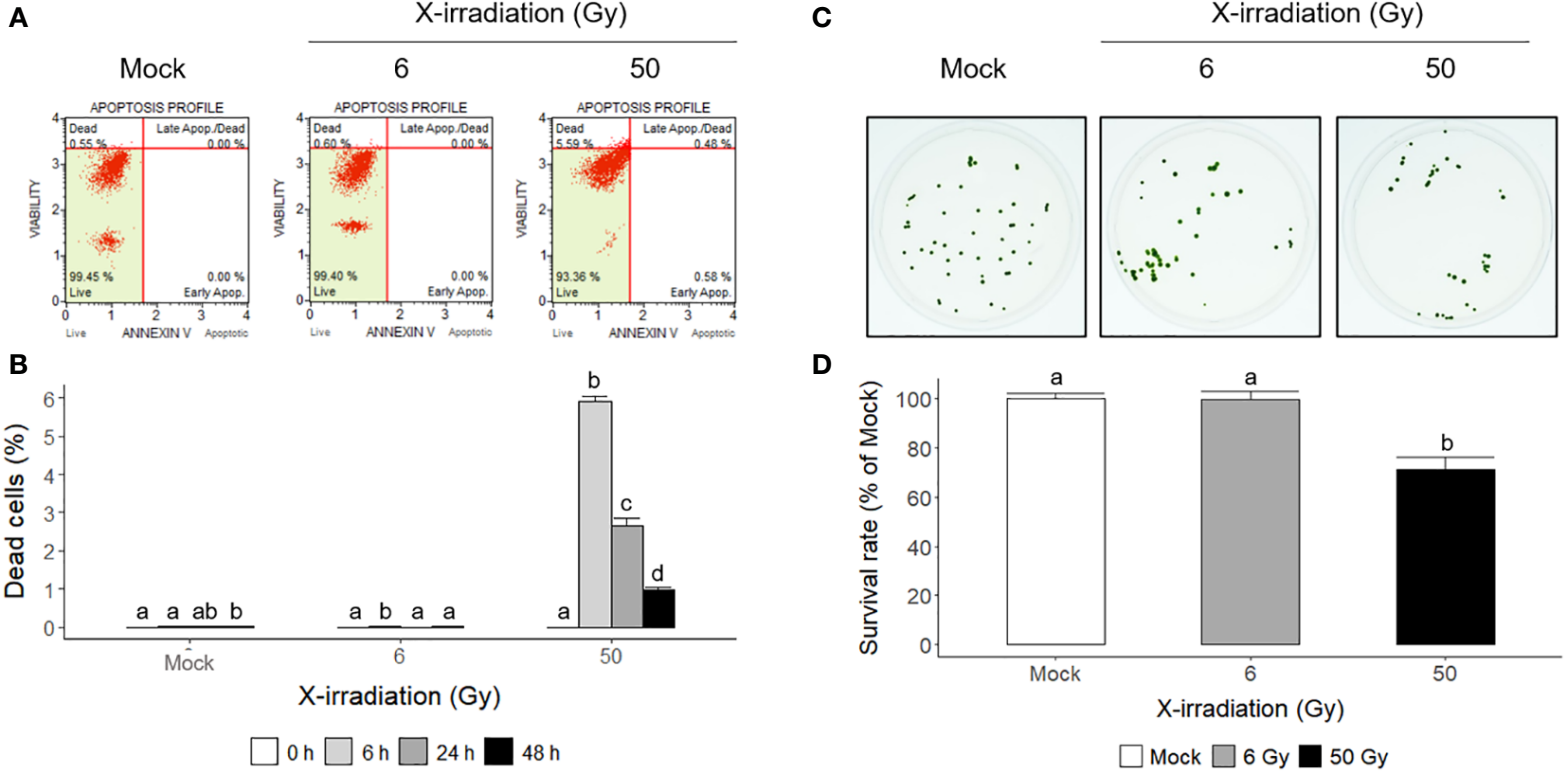
Apoptotic cell death and survival rates of *C. reinhardtii* cells after X-irradiation with 6 or 50 Gy. **(A, B)** The proportions of apoptotic and necrotic dead cells determined by flow cytometry at 6 h or during 48 h after X-irradiation with 6 or 50 Gy; **(C, D)** the survival rates of X-irradiated cells relative to the mock 7 days after X-irradiation with 6 or 50 Gy. In **(B, D)**, the data represent the mean ± standard error (SE) with *n* = 3 (5,000 cells for each) or *n* = 12 from three or four independent experiments, respectively. Different letters indicate significant differences at *p* < 0.05 (one-way analysis of variance followed by Tukey’s honestly significant difference test).

Flow cytometric analysis has been used as a rapid ecotoxicological tool to evaluate the effects of toxicity in *Chlamydomonas* cells using appropriate probes (Jamers et al., 2009). Propidium iodide (PI) and 7-aminoactinomycin D (7-AAD) are popular probes for quantifying the proportion of dead cells (Seed and Tomkins, 2016). In this study, mock and X-irradiated *C. reinhardtii* cells were subjected to double staining with Annexin V and 7-AAD to analyze apoptotic cell death. The cell death rate was significantly higher at 6 h after X-irradiation with 50 Gy and then decreased with time until 48 h, whereas X-irradiation with 6 Gy induced no significant difference in cell death compared to the mock treatment (**Figures 4A, B**). In addition, when the mock and X-irradiated *C. reinhardtii* cells were cultivated on solid TAP medium for 7 days, the relative cell survival rate decreased to approximately 30% in the 50 Gy irradiated group but showed no significant difference in the 6 Gy group (**Figures 4C, D**). These results demonstrate that the DDR gene expression induced after X-irradiation with 6 Gy, as shown in **Table 1** and **Figure 3**, does not represent fatal genotoxic stress responses, such as increased cell death and decreased survival rates. Although radiation-induced genotoxic stress or mutagenesis is generally accepted, the physiological responses of *C. reinhardtii* cells after X- or γ-irradiation are differentially modulated, depending on the absorbed dose.

### Differences in various metabolic activities of mock and X-irradiated *C. reinhardtii* cells

**Figure 5:**
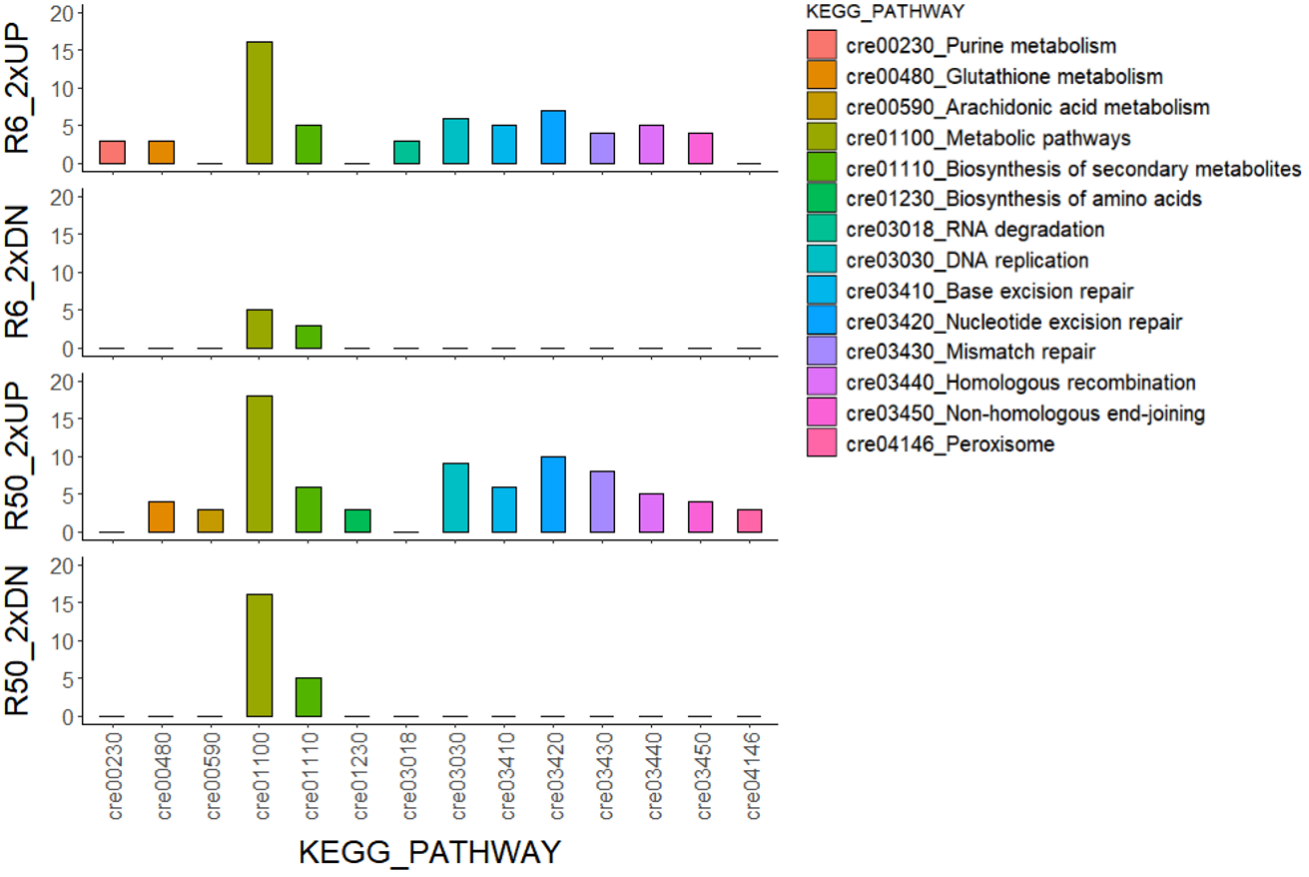
KEGG pathway analysis of genome-wide transcriptomic changes in *C. reinhardtii* cells after X- or γ-irradiation with two different doses. The numbers of common genes with a more than two-fold transcription change in both XR6 and GR6 (or XR50 and GR50) groups are 694 and 443 (or 824 and 541) for 2×UP and 2×DN, respectively. XR6 (GR6) and XR50 (GR50) represent X-rays (γ-rays) of 6 and 50 Gy, respectively.

**Figure 6:**
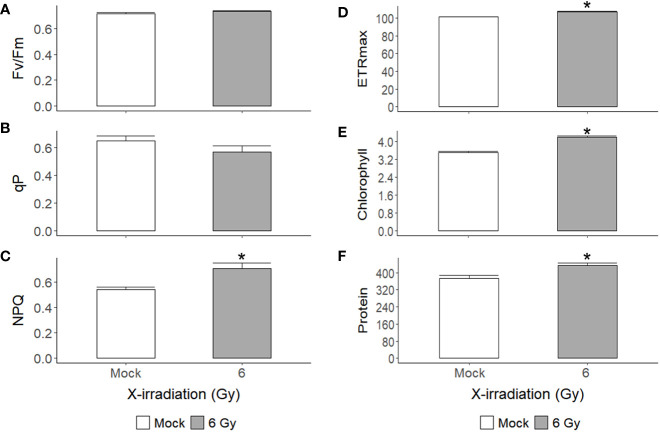
Changes in photosynthetic activity, chlorophyll, and protein contents of *C. reinhardtii* cells after repetitive X-irradiation with 6 Gy. **(A–D)** The chlorophyll fluorescence and quenching parameters Fv/Fm, qP, NPQ, and the maximal electron transport rate ETRmax were obtained and used to evaluate the photosynthetic activity of *C. reinhardtii* cells as described in the Materials and Methods section. **(E, F)** The chlorophyll and protein contents (μg) of *C. reinhardtii* cells (10^8^). Data represent the mean ± standard error (SE) with *n* = 9 from three independent experiments. Asterisks indicate significant differences between mock (white bars) and irradiated samples (gray bars) based on a two-sample independent *t*-test (*p* < 0.05).

**Figure 7:**
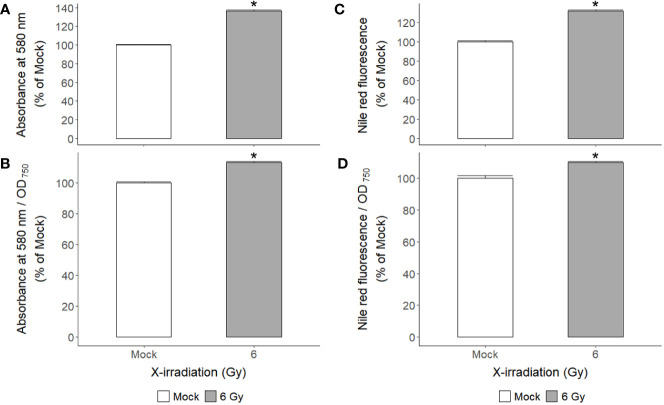
Changes in starch and lipid contents of *C. reinhardtii* cells after repetitive X-irradiation with 6 Gy. **(A)** The starch content was expressed relative to the mock by the absorbance at 580 nm after staining with the iodine reagent. **(C)** The lipid content was calculated relative to the mock by the signal at excitation 560 nm and emission 635 nm after staining with the Nile Red reagent. **(B, D)** The observed values were normalized against cell density (OD_750_) to get the starch or lipid content in the same number of cells and then expressed relative to the mock. Data represent the mean ± standard error (SE) with *n* = 9 from three independent experiments. Asterisks indicate significant differences between mock (white bars) and irradiated samples (gray bars) based on a two-sample independent *t*-test (*p* < 0.05).

X-irradiation at 6 Gy stimulated the growth of *C. reinhardtii* without inducing cell death or reducing survival (**Figures 1**, **2**, **4**). The gene ontology and KEGG pathway analyses of genome-wide transcriptomic changes after X- or γ-irradiation revealed substantial up-regulation in various metabolic pathways as well as DDR pathway (**Figure 5**). Therefore, to understand the favorable growth stimulation of X-irradiation with 6 Gy, we compared the mock and X-irradiated groups in terms of various metabolic activities. When the photosynthetic activity was evaluated by chlorophyll fluorescence analysis, the maximal photochemical efficiency of photosystem II (Fv/Fm), as well as the parameter for photochemical quenching, qP, were not substantially different between the mock and X-irradiated groups (**Figures 6A, B**). However, the parameter for non-photochemical quenching of chlorophyll fluorescence, NPQ, and the maximal electron transport rate of photosynthesis, ETRmax, were significantly increased in the latter group **(** **Figures 6C, D**). The changes in Fv/Fm, qP, and NPQ after X-irradiation were similar to those previously reported in some γ-irradiated groups of *C. reinhardtii* (Gomes et al., 2017), but the increase in ETRmax was first observed in this study. The chlorophyll and protein contents were 19% and 16% higher in the X-irradiated group than in the mock group, respectively (**Figures 6E, F**). In addition, the major algal metabolites such as starch and lipid were significantly increased by up to 136% and 130% (or 113% and 109% after normalization against cell density) in the X-irradiated group compared to the mock group, respectively (**Figure 7**). Correlation and discriminant analyses displayed differential correlations between the metabolic parameters including growth rate, photosynthetic activity, chlorophyll, protein, starch, and lipid contents with a distinct difference between the mock and X-irradiated groups of *C. reinhardtii* cells (**Supplementary Figure 4**). These results suggest that the growth stimulation of *C. reinhardtii* cells by X- or γ-irradiation with 6 Gy or less can be associated with the modulation of photosynthetic activity and enhancement of metabolic activities such as chlorophyll, protein, starch, and lipid synthesis. Our data demonstrate that X- or γ-irradiation with a certain range of relatively low doses can contribute to the improvement of algal biomass and metabolite yields *via* the growth stimulation of algal cells, despite the potential genotoxicity and oxidative stress.

### Enhanced growth stimulation of *C. reinhardtii* cells by cultivating with supplemented carbon source after repetitive X-irradiation with a relatively low dose, 6 Gy

**Figure 8:**
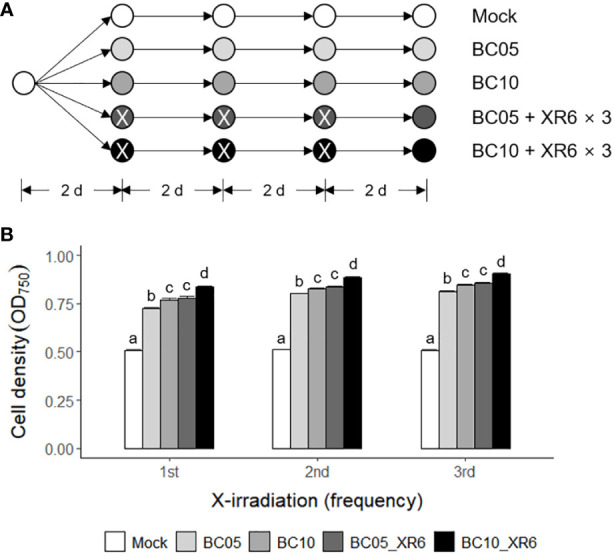
Synergistic growth stimulation of *C. reinhardtii* cells by cultivating in TAP medium containing sodium bicarbonate after repetitive X-irradiation with 6 Gy. BC05, BC10, and XR6 represent 5 and 10 mM NaHCO_3_ and X-rays of 6 Gy, respectively. **(A)** A schematic diagram of repetitive X-irradiation and treatment of NaHCO_3_. The absorbed dose of 6 Gy was repeatedly applied to the X-irradiated cells having an interval of 2 days. The cultivation of the mock was initiated at a cell density of 0.05 OD_750_. The irradiated and/or treated samples were subjected to post-irradiation and/or post-treatment cultivation with the equivalent volume of the mock to cumulate a difference in the growth rate after each X-irradiation and/or treatment of NaHCO_3_. **(B)** Cell density (OD_750_) of *C. reinhardtii* 2 days after single or repetitive X-irradiation. Data represent the mean ± standard error (SE) with *n* = 6 from two independent experiments. Different letters indicate significant differences at *p* < 0.05 (one-way analysis of variance followed by Tukey’s honestly significant difference test).

Carbon, nitrogen, and light are important factors in the growth and biomass yield of the green alga *Chlamydomonas* (Moon et al., 2013; Mortensen and Gislerod, 2015; Yang et al., 2018; Bialevich et al., 2022). Therefore, we investigated the combined effect of repetitive ‘low-dose’ X-irradiation and carbon source supplementation, as shown in **Figure 8A**. Although the standard TAP medium used in this study contained acetate as an organic carbon source, two concentrations (5 and 10 mM) of sodium bicarbonate (NaHCO_3_) as an inorganic carbon source were added to the TAP medium for growth stimulation. The growth rates of *C. reinhardtii* cells in the NaHCO_3_-supplemented TAP medium greatly increased in a concentration-dependent manner and significantly more increased when cultivated following X-irradiation at 6 Gy (**Figure 8B**). However, the enhanced growth stimulation after repetitive X-irradiation was not significant in TAP medium containing NaHCO_3_. When cultivated in 5 or 10 mM NaHCO_3_-supplemented TAP medium for 5 days after X-irradiation with 6 Gy, the growth rate of *C. reinhardtii* cells increased by 55% and 84% at 3 days after X-irradiation, respectively (**Supplementary Figure 5**). This growth stimulation was much stronger than the 37% or 42% induced by a single treatment with 5 or 10 mM NaHCO_3_ without X-irradiation. Taken together, the combination of X-irradiation at 6 Gy and NaHCO_3_ supplementation at 5 or 10 mM induces a synergistic effect for growth stimulation in *Chlamydomonas* cultivation.

### Radiation-sensitivity of different genotypes associated with a dose range of X-irradiation for growth stimulation of *C. reinhardtii* cells

**Figure 9:**
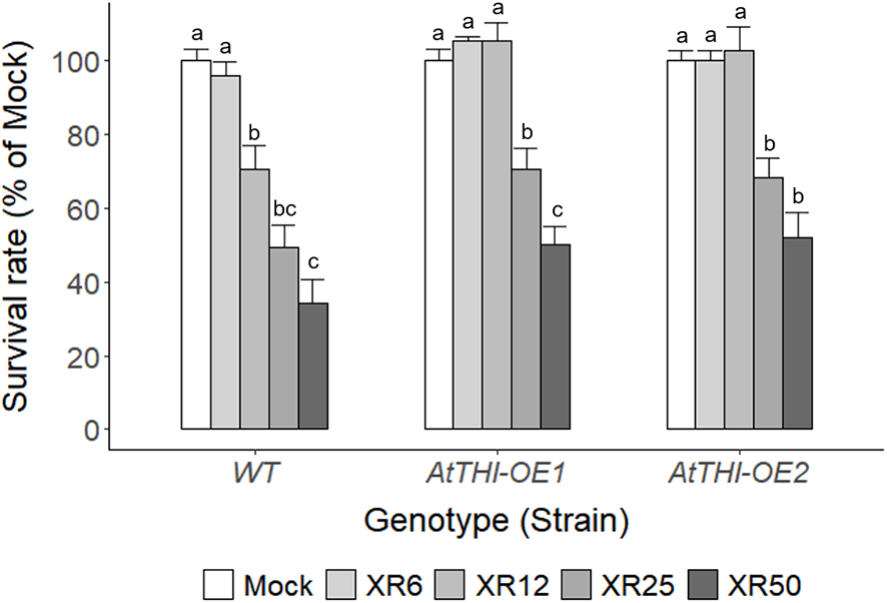
Differences in survival rates of wild type and transgenic lines of *C. reinhardtii* on solid TAP medium after X-irradiation with different doses. The survival rates of X-irradiated cells relative to the mock were calculated by counting the number of colonies on the solid TAP medium 7 days after X-irradiation with 6, 12, 25, or 50 Gy. WT, wild type. XR6, XR12, XR25, and XR50 represent X-rays of 6, 12, 25, and 50 Gy, respectively. Data represent the mean ± standard error (SE) with *n* = 6 from two independent experiments. Different letters indicate significant differences at *p* < 0.05 (one-way analysis of variance followed by Tukey’s honestly significant difference test).

Even in the same species, general stress responses are expected to differ according to individual genotypes. As *Chlamydomonas* is a widely used algal platform for recombinant protein production (Kumar et al., 2013; Rasala and Mayfield, 2015; Doron et al., 2016; Ma et al., 2022), the applicability of radiation-induced growth stimulation is worth investigating in transgenic *Chlamydomonas* producing recombinant proteins. Therefore, two transgenic *C. reinhardtii* lines (*AtTHI-OE1* and *AtTHI-OE2*), which overexpress *AtTHI2.1* gene encoding *Arabidopsis* antibacterial proteins, thionins, were randomly selected and compared with wild type (WT) in the cell growth and survival rates after X-irradiation at a dose range of 6–50 Gy (**Supplementary Figure 6** and **Figure 9**). The cell growth rate after X-irradiation was distinctly different between WT and *AtTHI-OE1* or *AtTHI-OE2* lines, showing the growth stimulation in the 6 Gy group for WT and in the 12 Gy group for *AtTHI-OE1* and *AtTHI-OE2* (**Supplementary Figure 6B**). When the mock and X-irradiated *C. reinhardtii* cells were cultivated for 7 days on solid TAP medium, the relative cell survival rate was higher in the *AtTHI-OE1* and *AtTHI-OE2* lines than in the WT after X-irradiation with 25 or 50 Gy (**Figure 9**). The survival rate started to decrease in the 6 Gy group for WT and the 12 Gy group for *AtTHI-OE1* and *AtTHI-OE2* in a dose- and genotype-dependent manner. In addition, the expression levels of two DDR genes *CrRPA70A* and *CrRAD51* were generally lower in the *AtTHI-OE1* and *AtTHI-OE2* lines than in the WT after X-irradiation (**Supplementary Figure 6C**). These results suggest that the radiation sensitivity of *C. reinhardtii* cells differs among genotypes and may be closely associated with an optimal dose range of X-irradiation for growth stimulation.

### Correlation between ROS accumulation and radiation-induced growth stimulation of *C. reinhardtii* cells after X-irradiation with 6 Gy

**Figure 10:**
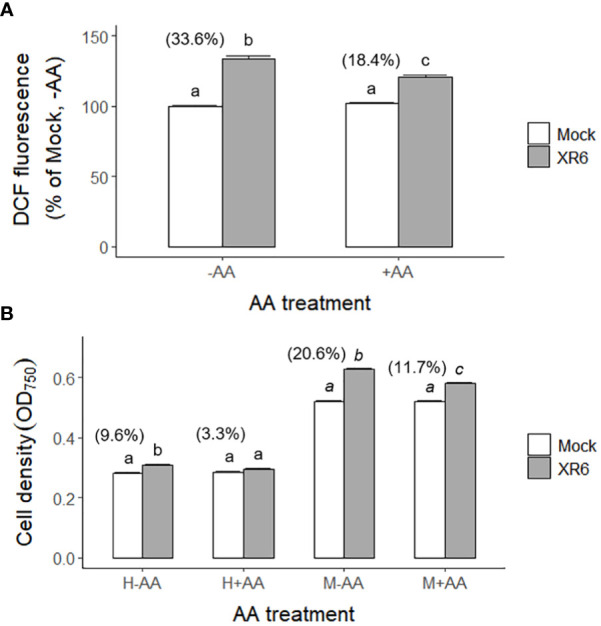
Changes in ROS levels and growth rates of *C. reinhardtii* cells by treatment of ascorbic acid (AA) following X-irradiation with 6 Gy. XR6 represents X-rays of 6 Gy. **(A)** The ROS level was calculated relative to the mock without AA treatment by the DCF signal at excitation 480 nm and emission 530 nm as described in the Materials and Methods section. **(B)** Cell density (OD_750_) of *C. reinhardtii* 2 days after the second repetitive X-irradiation. Cells were incubated without or with 1 mM AA for 1 h at 25°C after X-irradiation, and were subjected to heterotrophic (H) cultivation in darkness or mixotrophic (M) cultivation under light for 2 days. Data represent the mean ± standard error (SE) with *n* = 6 from two independent experiments. Different letters indicate significant differences at *p* < 0.05 (one-way analysis of variance followed by Tukey’s honestly significant difference test). Digits in parentheses are percent differences in average between mock and irradiated cells.

Ionizing radiation generates various types of ROS such as hydrogen peroxide (H_2_O_2_) and hydroxyl radical (•OH) in a dose-dependent manner *via* water radiolysis (Lee et al., 2009). Especially, H_2_O_2_ is the main ROS to mediate both oxidative damage and signaling in plants and algae (Quan et al., 2008; Battah et al., 2015; Niu and Liao, 2016; Fal et al., 2022). X-irradiation at a dose range of 3–100 Gy induced a biphasic increase of ROS in a dose- and dose rate-dependent manner including the relatively low doses of 3 and 6 Gy (**Supplementary Figure 7**). In contrast, the *in vivo* accumulation of radiation-induced ROS in *C. reinhardtii* cells after X-irradiation with 6 Gy decreased from 33.6% to 18.4% by a subsequent treatment of ascorbic acid (AA), a ROS scavenger (**Figure 10A**). Therefore, we investigated the effect of ROS accumulation on the radiation-induced growth stimulation of *C. reinhardtii* cells. The radiation-induced growth stimulation of *C. reinhardtii* cells after X-irradiation with 6 Gy significantly decreased from 9.6% to 3.3% or from 20.6% to 11.7% by AA treatment before heterotrophic or mixotrophic cultivation, respectively (**Figure 10B**). These results suggest that radiation-induced growth stimulation could be closely associated with ROS accumulation and signaling.

## Discussion

Ionizing radiation has been used as a potent mutagen for *Chlamydomonas* mutation breeding (Baek et al., 2016; Koo et al., 2017b). It is also regarded as an oxidative stress inducer that generates ROS *via* water radiolysis (Lee et al., 2009; Gomes et al., 2017; Singh et al., 2022). Gamma irradiation at a dose range of 0.5–4 kGy inhibited the growth of *Chlorella sorokiniana* cells in a dose-dependent manner (Singh et al., 2022). In contrast, this study demonstrated that X- or γ-irradiation at a relatively low dose below 10 Gy substantially increased the growth of *C. reinhardtii* cells rather than cell death (**Figures 1**, **2**, **4**). The counterintuitive dose-response was previously reported in red pepper (*Capsicum annuum* L.) plants, enhancing growth and stress resistance after γ-irradiation with 2 or 4 Gy (Kim et al., 2005). In addition, γ-irradiation with a much lower dose of 0.1 Gy decreased oncogene-induced malignant transformation in human cells through the reduction of ROS (Kim et al., 2015b). The bioeffects of low-dose or low-dose-rate ionizing radiation are controversial and depend on, among others, animal genetic background, age, sex, nature of radiation exposure, type of radiation, and the combination of radiation with other toxic agents in animal models (Tang et al., 2017). However, our results imply that the growth stimulatory effect of ionizing radiation at relatively low doses can be substantiated in algal cells. Although ionizing radiation is a potent mutagenic agent for algal breeding, it may also be an efficient elicitor to facilitate algal cultivation.

The growth of *C. reinhardtii* cells was substantially enhanced by X-irradiation at 6 Gy but was inhibited by treatment with 50 Gy (**Figure 1**). In comparison, X-irradiation at doses of 6 and 50 Gy significantly increased both starch and lipid contents in a dose-dependent manner (**Figure 7** and unpublished data). The effects of X-irradiation with 6 Gy were comparable to those of the synthetic compounds WD10784 and WD30030, which increased starch and/or lipid synthesis in *Chlamydomonas* cells without restricting growth or biomass yield (Wase et al., 2019). Metabolic remodeling of synthetic inducers is associated with increased substrate availability for starch and lipid synthesis, but that of ionizing radiation at relatively low doses has rarely been elucidated in photosynthetic organisms such as plants and algae. The DEGs in *C. reinhardtii* cells after X- or γ-irradiation with 6 or 50 Gy were mainly associated with DDR in a dose-dependent manner (**Table 1** and **Figure 6**). The transcriptomic changes in *C. reinhardtii* cells after X- or γ-irradiation with 6 or 50 Gy were much less than those in *Arabidopsis* plants after γ-irradiation at 200 Gy (Kim et al., 2013). Probably due to the limited numbers or insufficient annotations of common genes with a more than two-fold transcription change in both the X- and γ-irradiated groups, the DEGs were substantially up-regulated in various metabolic pathways as well as DDR pathway, but were difficult to be causally associated with the differences in growth and metabolic activities between the 6 and 50 Gy groups (**Figures 5**). However, because relatively low doses below 10 Gy accumulated ROS in *C. reinhardtii* cells (Gomes et al., 2017), the overall transcriptomic changes caused by mild oxidative stress may partly contribute to the altered metabolic activities and growth stimulation. The radiation-induced growth stimulation of *C. reinhardtii* cells may be associated with enhanced metabolic activities, such as photosynthesis, chlorophyll, and protein synthesis. Considering the dynamic metabolic carbon flow that modulates the relative proportions of starch and lipids in a reverse relationship (Sato and Toyoshima, 2021), the simultaneous enhancement of starch and lipid synthesis after X-irradiation with 6 Gy is likely to be attributed to the metabolic alterations associated with growth stimulation.

The chlorophyll fluorescence parameters (NPQ and ETRmax) for photosynthetic activity and chlorophyll content increased simultaneously and significantly in *C. reinhardtii* cells after X-irradiation at 6 Gy (**Figures 6C, D**). In contrast, γ-irradiation at doses of approximately 6 Gy was previously reported to increase NPQ but decrease qP and ETR (Gomes et al., 2017). This difference may be due to the high-salt growth medium and the much lower dose rate of γ-irradiation, which were used in the latter study. When *Arabidopsis* plants were gamma-irradiated with total doses of 3.9, 6.7, 14.8, and 58.8 Gy at dose rates of 23.3, 40.4, 89.2, and 353.1 mGy h^-1^, they displayed a decrease in NPQ but an increase in ETRmax (Vanhoudt et al., 2014). However, after γ-irradiation at a dose rate of 50 Gy h^-1^ for 4 h, NPQ was found to consistently decrease with ETRmax or the performance index of photosynthesis in various plant species, including *Arabidopsis*, Chinese cabbage, cucumber, tomato, lettuce, and red pepper (Moon et al., 2008; Kim et al., 2011). Therefore, the increased NPQ in *C. reinhardtii* cells after X- or γ-irradiation with approximately 6 Gy does not imply a substantial decrease in photosynthetic activity. Rather, this may be a photoprotective mechanism for the enhanced ETRmax due to increased chlorophyll content. Taken together, the metabolic parameters including growth rate, photosynthetic activity, chlorophyll, protein, starch, and lipid contents seem to be correlated but not in a causal relationship (**Supplementary Figure 4**). In *Chlorella sorokiniana* cells, γ-irradiation at a dose range of 0.5–4 kGy decreased growth, chlorophyll content, and photosynthetic efficiency in a dose-dependent manner but increased ROS and lipid accumulation (Singh et al., 2022). The growth stimulatory effects of X- or γ-irradiation with a certain range of relatively low doses in algal cells could be associated with enhanced metabolic activities but not explained by a causal relationship between them.

In the commercial cultivation of *Chlamydomonas* cells, nutrient composition and culture conditions are critical factors affecting cell growth and biomass yield (Fields et al., 2018; Yang et al., 2018; Bialevich et al., 2022). In this study, we demonstrated that radiation-induced growth stimulation of *C. reinhardtii* cells was more strongly enhanced through batch cultivation in fresh TAP medium supplemented with an inorganic carbon source, NaHCO_3_, after single or repetitive X-irradiation with 6 Gy (**Figure 8** and **Supplementary Figure 5**). In addition, the growth stimulation of *C. reinhardtii* cells after X-irradiation with 6 Gy was different in minimal TP, reused TAP, or nitrogen source-supplemented medium (unpublished data). This implies that different nutrient compositions could modulate the low-dose growth stimulation of *Chlamydomonas* cells. Therefore, although the early growth stimulation of red pepper plants after γ-irradiation with 2 or 4 Gy was not evaluated at later developmental stages (Kim et al., 2004; Kim et al., 2005), the culture conditions of unicellular green algae such as *Chlamydomonas* can be more easily optimized for low-dose X- or γ-irradiation to substantially increase growth and biomass yield.

As the low-dose radiation-induced bioeffects are known to differ by, among others, the genetic background and age in animal models (Tang et al., 2017), the genotype and growth phase of *C. reinhardtii* cells were considered critical factors affecting the radiation-induced stimulatory effects throughout the present study. Algae have been used as a potential platform to produce recombinant proteins through nuclear and chloroplast transformations (Rasala and Mayfield, 2015; Doron et al., 2016). The integration of transgenes in algal genomes and their expression may affect low-dose radiation-induced stimulatory effects, such as enhanced growth and metabolic activities, by establishing a new genotype. In this study, the radiation-induced growth stimulation of *C. reinhardtii* cells was differentially observed in the 6 Gy group for WT but in the 12 Gy group for two transgenic lines, *AtTHI-OE1* and *AtTHI-OE2*, which was associated with the differences in the relative cell survival rate and DDR gene expression between the two groups (**Supplementary Figure 6** and **Figure 9**). Therefore, genotype-dependent radiation sensitivity may determine a specific dose range for the stimulatory effects of ionizing radiation in *C. reinhardtii* cells. In addition, an epigenetic transgene silencing pathway has recently been elucidated in the green alga *Chlamydomonas* (Neupert et al., 2020). Epigenetic regulatory mechanisms need to be explored for elucidating low-dose radiation-induced stimulatory effects in nuclear-transformed transgenic lines with transgene silencing or growth inhibition.

The radiation-induced growth stimulation of *C. reinhardtii* cells may be explained by epigenetic regulatory mechanisms for stress priming, which have been reported in plant stress responses (Kim, 2021). Ionizing radiation influences the expression of DDR genes *via* chromatin remodeling and epigenetic regulation (Mondal et al., 2016; Kim, 2019). It can induce oxidative stress responses by generating ROS and activating various defense mechanisms for stress priming in plant and algal cells (Kim et al., 2005; Gomes et al., 2017). In fact, this study demonstrated that the radiation-induced growth stimulation of *C. reinhardtii* cells after X-irradiation with 6 Gy was closely associated with ROS accumulation and significantly inhibited by treatment of AA, a ROS scavenger (**Supplementary Figure 7** and **Figure 10**). Therefore, X- or γ-irradiation with a certain range of relatively low doses seems to induce stress memory or priming effects as an elicitor, contributing to enhanced growth and metabolic activities *via* ROS-mediated metabolic remodeling. The stimulatory effects of low-dose radiation in *C. reinhardtii* cells imply that potential DNA damages from genotoxicity may not necessarily determine the fate of irradiated cells. This hypothesis is also supported by the fact that treatment with a ROS scavenger inhibited metabolic remodeling for lipid accumulation in the γ-irradiated cells of *Chlorella sorokiniana* (Singh et al., 2022). In addition, since radiation-induced metabolic disturbances such as chlorosis or delayed senescence in *Arabidopsis* plants differed according to the developmental stage subjected to γ-irradiation with 200 Gy (Kim et al., 2009; Kim et al., 2011), the different sensitivities of individual plants or cells to ionizing radiation may be crucial in determining radiation-induced damage or priming effects. We suggest ROS-mediated stress priming or memory as one of the mechanisms that enable the counterintuitive stimulatory effects of ionizing radiation on *C. reinhardtii* cells. This phenomenon of low-dose radiation is also recognized as a bystander effect and adaptive response in animal systems (Bonner, 2003).

## Conclusion

In this study, we demonstrated that ionizing radiation within a certain dose range determined by genotype-dependent radiation sensitivity could act as an elicitor to induce growth stimulation and enhance metabolic activities, including photosynthesis, chlorophyll, protein, starch, and lipid synthesis in *Chlamydomonas* cells. This is the first report to reveal the counterintuitive beneficial effects of a genotoxic and abiotic stress factor, ionizing radiation, in the unicellular algal organism *Chlamydomonas*. The data obtained from the unicellular *Chlamydomonas* system were evaluated as new practical examples of low-dose or low-dose-rate radiation-induced positive bioeffects, which have been reported mainly in plant and animal systems. However, many low-dose radiation-induced bioeffects, including our results, remain to be further elucidated regarding detailed causal mechanisms, such as epigenetic regulation.

## Data availability statement

The original contributions presented in the study are publicly available. This data can be found here: NCBI, GSE218435.

## Author contributions

J-HK conceived, designed, and supervised the study. J-HK, SKD, and KH performed the experiments and generated the raw data. J-HK and SKD analyzed, interpreted, and visualized the data. J-HK wrote the manuscript and SKD, BYC, SSL, and SL critically reviewed the manuscript. All authors contributed to the article and approved the submitted version.

## References

[B1] Baek J. Choi J. I. Park H. Lim S. Park S. J. (2016). Isolation and proteomic analysis of a *Chlamydomonas reinhardtii* mutant with enhanced lipid production by the gamma irradiation method. J. Microbiol. Biotechnol. 26, 2066–2075. doi: 10.4014/jmb.1605.05057 27586532

[B2] Battah M. El-Ayoty Y. Abomohra A. E.-F. El-Ghany S. A. Esmael A. (2015). Effect of Mn^2+^, Co^2+^ and H_2_O_2_ on biomass and lipids of the green microalga *Chlorella vulgaris* as a potential candidate for biodiesel production. Ann. Microbiol. 65, 155–162. doi: 10.1007/s13213-014-0846-7

[B3] Bialevich V. Zachleder V. Bisova K. (2022). The effect of variable light source and light intensity on the growth of three algal species. Cells 11, 1293. doi: 10.3390/cells11081293 35455972 PMC9028354

[B4] Bischof K. Hanelt D. Wiencke C. (2000). Effects of ultraviolet radiation on photosynthesis and related enzyme reactions of marine macroalgae. Planta 211, 555–562. doi: 10.1007/s004250000313 11030555

[B5] Bonner W. M. (2003). Low-dose radiation: thresholds, bystander effects, and adaptive responses. Proc. Natl. Acad. Sci. U.S.A. 100, 4973–4975. doi: 10.1073/pnas.1031538100 12704228 PMC154280

[B6] Bradford M. M. (1976). A rapid and sensitive method for the quantitation of microgram quantities of protein utilizing the principle of protein-dye binding. Anal. Biochem. 72, 248–254. doi: 10.1016/0003-2697(76)90527-3 942051

[B7] Cakmak T. Angun P. Demiray Y. E. Ozkan A. D. Elibol Z. Tekinay T. (2012). Differential effects of nitrogen and sulfur deprivation on growth and biodiesel feedstock production of *Chlamydomonas reinhardtii* . Biotechnol. Bioeng. 109, 1947–1957. doi: 10.1002/bit.24474 22383222

[B8] Charoonnart P. Worakajit N. Zedler J. A. Z. Meetam M. Robinson C. Saksmerprome V. (2019). Generation of microalga *Chlamydomonas reinhardtii* expressing shrimp antiviral dsRNA without supplementation of antibiotics. Sci. Rep. 9, 3164. doi: 10.1038/s41598-019-39539-x 30816201 PMC6395707

[B9] Doron L. Segal N. Shapira M. (2016). Transgene expression in microalgae-from tools to applications. Front. Plant Sci. 7, 505. doi: 10.3389/fpls.2016.00505 27148328 PMC4840263

[B10] Fal S. Aasfar A. Rabie R. Smouni A. Arroussi H. E. (2022). Salt induced oxidative stress alters physiological, biochemical and metabolomic responses of green microalga *Chlamydomonas reinhardtii* . Heliyon 8, e08811. doi: 10.1016/j.heliyon.2022.e08811 35118209 PMC8792077

[B11] Fan J. Cui Y. Wan M. Wang W. Li Y. (2014). Lipid accumulation and biosynthesis genes response of the oleaginous *Chlorella pyrenoidosa* under three nutrition stressors. Biotechnol. Biofuels 7, 17. doi: 10.1186/1754-6834-7-17 24479413 PMC3916312

[B12] Fan J. Zheng L. (2017). Acclimation to NaCl and light stress of heterotrophic *Chlamydomonas reinhardtii* for lipid accumulation. J. Biosci. Bioeng. 124, 302–308. doi: 10.1016/j.jbiosc.2017.04.009 28483385

[B13] Ferrante P. Diener D. R. Rosenbaum J. L. Giuliano G. (2011). Nickel and low CO_2_-controlled motility in *Chlamydomonas* through complementation of a paralyzed flagella mutant with chemically regulated promoters. BMC Plant Biol. 11, 22. doi: 10.1186/1471-2229-11-22 21266063 PMC3038898

[B14] Fields F. J. Ostrand J. T. Mayfield S. P. (2018). Fed-batch mixotrophic cultivation of *Chlamydomonas reinhardtii* for high-density cultures. Algal Res. 33, 109–117. doi: 10.1016/j.algal.2018.05.006

[B15] Gomes T. Xie L. Brede D. Lind O.-C. Solhaug K. A. Salbu B.. (2017). Sensitivity of the green algae *Chlamydomonas reinhardtii* to gamma radiation: Photosynthetic performance and ROS formation. Aquat. Toxicol. 183, 1–10. doi: 10.1016/j.aquatox.2016.12.001 27978482

[B16] Harris E. H. (1989). The chlamydomonas sourcebook: A comprehensive guide to biology and laboratory use (San Diego, CA: Academic Press). 10.1126/science.246.4936.1503-a 17756009

[B17] Harris E. H. (2001). *Chlamydomonas* as a model organism. Annu. Rev. Plant Physiol. Plant Mol. Biol. 52, 363–406. doi: 10.1146/annurev.arplant.52.1.363 11337403

[B18] Huang D. W. Sherman B. T. Lempicki R. A. (2009). Systematic and integrative analysis of large gene lists using DAVID bioinformatics resources. Nat. Protoc. 4, 44–57. doi: 10.1038/nprot.2008.211 19131956

[B19] Jamers A. Lenjou M. Deraedt P. Bockstaele D. V. Blust R. Coen W. D. (2009). Flow cytometric analysis of the cadmium-exposed green alga *Chlamydomonas reinhardtii* (*Chlorophyceae*). Eur. J. Phycol. 44, 541–550. doi: 10.1080/09670260903118214

[B20] Jung S. Koo K. M. Ryu J. Baek I. Kwon S. J. Kim J. B.. (2021). Overexpression of phosphoribosyl pyrophosphate synthase enhances resistance of *Chlamydomonas* to ionizing radiation. Front. Plant Sci. 12, 719846. doi: 10.3389/fpls.2021.719846 34512699 PMC8427504

[B21] Kim J. H. (2019). Chromatin remodeling and epigenetic regulation in plant DNA damage repair. Int. J. Mol. Sci. 20, 4093. doi: 10.3390/ijms20174093 31443358 PMC6747262

[B22] Kim J. H. (2021). Multifaceted chromatin structure and transcription changes in plant stress response. Int. J. Mol. Sci. 22, 2013. doi: 10.3390/ijms22042013 33670556 PMC7922328

[B23] Kim J.-H. Baek M.-H. Chung B. Y. Wi S. G. Kim J.-S. (2004). Alterations in the photosynthetic pigments and antioxidant machineries of red pepper (*Capsicum annuum* l.) seedlings from gamma-irradiated seeds. J. Plant Biol. 47, 314–321. doi: 10.1007/BF03030546

[B24] Kim J.-H. Chung B. Y. Kim J.-S. Wi S. G. (2005). Effects of *in planta* gamma-irradiation on growth, photosynthesis, and antioxidative capacity of red pepper (*Capsicum annuum* l.) plants. J. Plant Biol. 48, 47–56. doi: 10.1007/BF03030564

[B25] Kim J. H. Hwangbo K. Lee E. Dubey S. K. Chung M. S. Chung B. Y.. (2021). Application of gamma ray-responsive genes for transcriptome-based phytodosimetry in rice. Plants (Basel) 10, 968. doi: 10.3390/plants10050968 34067996 PMC8152246

[B26] Kim J.-H. Kim J. E. Lee M. H. Lee S. W. Cho E. J. Chung B. Y. (2013). Integrated analysis of diverse transcriptomic data from *Arabidopsis* reveals genetic markers that reliably and reproducibly respond to ionizing radiation. Gene 518, 273–279. doi: 10.1016/j.gene.2013.01.027 23376455

[B27] Kim R. K. Kim M. J. Seong K. M. Kaushik N. Suh Y. Yoo K. C.. (2015b). Beneficial effects of low dose radiation in response to the oncogenic KRAS induced cellular transformation. Sci. Rep. 5, 15809. doi: 10.1038/srep15809 26515758 PMC4626770

[B28] Kim D. Langmead B. Salzberg S. L. (2015a). HISAT: A fast spliced aligner with low memory requirements. Nat. Methods 12, 357–360. doi: 10.1038/nmeth.3317 25751142 PMC4655817

[B29] Kim J.-H. Lee M. H. Moon Y. R. Kim J.-S. Wi S. G. Kim T. H.. (2009). Characterization of metabolic disturbances closely linked to the delayed senescence of *Arabidopsis* leaves after γ irradiation. Environ. Exp. Bot. 67, 363–371. doi: 10.1016/j.envexpbot.2009.07.001

[B30] Kim J.-H. Moon Y. R. Lee M. H. Kim J. H. Wi S. G. Park B. J.. (2011). Photosynthetic capacity of *Arabidopsis* plants at the reproductive stage tolerates gamma irradiation. J. Radiat. Res. 52, 441–449. doi: 10.1269/jrr.10157 21785233

[B31] Kim J. H. Ryu T. H. Lee S. S. Lee S. Chung B. Y. (2019). Ionizing radiation manifesting DNA damage response in plants: An overview of DNA damage signaling and repair mechanisms in plants. Plant Sci. 278, 44–53. doi: 10.1016/j.plantsci.2018.10.013 30471728

[B32] Kliphuis A. M. Klok A. J. Martens D. E. Lamers P. P. Janssen M. Wijffels R. H. (2012). Metabolic modeling of *Chlamydomonas reinhardtii*: energy requirements for photoautotrophic growth and maintenance. J. Appl. Phycol. 24, 253–266. doi: 10.1007/s10811-011-9674-3 22427720 PMC3289792

[B33] Koo K. M. Jung S. Kim J. B. Kim S. H. Kwon S. J. Jeong W. J.. (2017a). Effect of ionizing radiation on the DNA damage response in *Chlamydomonas reinhardtii* . Genes Genomics 39, 63–75. doi: 10.1007/s13258-016-0472-9

[B34] Koo K. M. Jung S. Lee B. S. Kim J. B. Jo Y. D. Choi H. I.. (2017b). The mechanism of starch over-accumulation in *Chlamydomonas reinhardtii* high-starch mutants identified by comparative transcriptome analysis. Front. Microbiol. 8, 858. doi: 10.3389/fmicb.2017.00858 28588557 PMC5440458

[B35] Kumar A. Falcao V. R. Sayre R. T. (2013). Evaluating nuclear transgene expression systems in *Chlamydomonas reinhardtii* . Algal Res. 2, 321–332. doi: 10.1016/j.algal.2013.09.002

[B36] Lee M. H. Moon Y. R. Chung B. Y. Kim J.-S. Lee K.-S. Cho J.-Y.. (2009). Practical use of chemical probes for reactive oxygen species produced in biological systems by γ-irradiation. Radiat. Phys. Chem. 78, 323–327. doi: 10.1016/j.radphyschem.2009.03.001

[B37] Lichtenthaler H. K. (1987). Chlorophylls and carotenoids: Pigments of photosynthetic biomembranes. Methods Enzymol. 148, 350–382. doi: 10.1016/0076-6879(87)48036-1

[B38] Li Y. Han D. Hu G. Sommerfeld M. Hu Q. (2010). Inhibition of starch synthesis results in overproduction of lipids in *Chlamydomonas reinhardtii* . Biotechnol. Bioeng. 107, 258–268. doi: 10.1002/bit.22807 20506159

[B39] Li S. Ji L. Chen C. Zhao S. Sun M. Gao Z.. (2020). Efficient accumulation of high-value bioactive substances by carbon to nitrogen ratio regulation in marine microalgae *Porphyridium purpureum* . Bioresour. Technol. 309, 123362. doi: 10.1016/j.biortech.2020.123362 32305848

[B40] Liu W. Saint D. A. (2002). A new quantitative method of real time reverse transcription polymerase chain reaction assay based on simulation of polymerase chain reaction kinetics. Anal. Biochem. 302, 52–59. doi: 10.1006/abio.2001.5530 11846375

[B41] Ma K. Deng L. Wu H. Fan J. (2022). Towards green biomanufacturing of high-value recombinant proteins using promising cell factory: *Chlamydomonas reinhardtii* chloroplast. Bioresour. Bioprocess. 9, 83. doi: 10.1186/s40643-022-00568-6 PMC10992328 38647750

[B42] Merchant S. S. Prochnik S. E. Vallon O. Harris E. H. Karpowicz S. J. Witman G. B.. (2007). The *Chlamydomonas* genome reveals the evolution of key animal and plant functions. Science 318, 245–250. doi: 10.1126/science.1143609 17932292 PMC2875087

[B43] Mondal S. Go Y. S. Lee S. S. Chung B. Y. Kim J. H. (2016). Characterization of histone modifications associated with DNA damage repair genes upon exposure to gamma rays in *Arabidopsis* seedlings. J. Radiat. Res. 57, 646–654. doi: 10.1093/jrr/rrw077 27534791 PMC5137295

[B44] Moon Y. R. Kim J.-H. Lee M. H. Kim J.-S. Chung B. Y. (2008). Thermal dissipation of excess light in *Arabidopsis* leaves is inhibited after gamma-irradiation. J. Plant Biol. 51, 52–57. doi: 10.1007/BF03030741

[B45] Moon M. Kim C. W. Park W.-K. Yoo G. Choi Y.-E. Yang J.-W. (2013). Mixotrophic growth with acetate or volatile fatty acids maximizes growth and lipid production in *Chlamydomonas reinhardtii* . Algal Res. 2, 352–357. doi: 10.1016/j.algal.2013.09.003

[B46] Mortensen L. M. Gislerod H. R. (2015). The growth of *Chlamydomonas reinhardtii* as influenced by high CO_2_ and low O_2_ in flue gas from a silicomanganese smelter. J. Appl. Phycol. 27, 633–638. doi: 10.1007/s10811-014-0357-8 25866444 PMC4387248

[B47] Neupert J. Gallaher S. D. Lu Y. Strenkert D. Segal N. Barahimipour R.. (2020). An epigenetic gene silencing pathway selectively acting on transgenic DNA in the green alga *Chlamydomonas* . Nat. Commun. 11, 6269. doi: 10.1038/s41467-020-19983-4 33293544 PMC7722844

[B48] Niu L. Liao W. (2016). Hydrogen peroxide signaling in plant development and abiotic responses: Crosstalk with nitric oxide and calcium. Front. Plant Sci. 7, 230. doi: 10.3389/fpls.2016.00230 26973673 PMC4777889

[B49] Park J. J. Wang H. Gargouri M. Deshpande R. R. Skepper J. N. Holguin F. O.. (2015). The response of *Chlamydomonas reinhardtii* to nitrogen deprivation: a systems biology analysis. Plant J. 81, 611–624. doi: 10.1111/tpj.12747 25515814

[B50] Quan L. J. Zhang B. Shi W. W. Li H. Y. (2008). Hydrogen peroxide in plants: a versatile molecule of the reactive oxygen species network. J. Integr. Plant Biol. 50, 2–18. doi: 10.1111/j.1744-7909.2007.00599.x 18666947

[B51] Rasala B. A. Mayfield S. P. (2015). Photosynthetic biomanufacturing in green algae; production of recombinant proteins for industrial, nutritional, and medical uses. Photosynth. Res. 123, 227–239. doi: 10.1007/s11120-014-9994-7 24659086

[B52] R Core Team (2022). R: A language and environment for statistical computing (Vienna, Austria: R Foundation for Statistical Computing). Available at: https://www.R-project.org/.

[B53] Rstudio Team (2022). RStudio: Integrated development environment for r (PBC, Boston, MA: RStudio, Inc.). Available at: http://www.rstudio.com/.

[B54] Ryu T. H. Kim J. K. Kim J. I. Kim J. H. (2018). Transcriptome-based biological dosimetry of gamma radiation in *Arabidopsis* using DNA damage response genes. J. Environ. Radioact. 181, 94–101. doi: 10.1016/j.jenvrad.2017.11.007 29128690

[B55] Sasso S. Stibor H. Mittag M. Grossman A. R. (2018). From molecular manipulation of domesticated *Chlamydomonas reinhardtii* to survival in nature. Elife 7, e39233. doi: 10.7554/eLife.39233.011 30382941 PMC6211829

[B56] Sato N. Toyoshima M. (2021). Dynamism of metabolic carbon flow of starch and lipids in *Chlamydomonas debaryana* . Front. Plant Sci. 12, 646498. doi: 10.3389/fpls.2021.646498 33868347 PMC8047662

[B57] Scranton M. A. Ostrand J. T. Fields F. J. Mayfield S. P. (2015). *Chlamydomonas* as a model for biofuels and bio-products production. Plant J. 82, 523–531. doi: 10.1111/tpj.12780 25641390 PMC5531182

[B58] Seed C. E. Tomkins J. L. (2016). Flow cytometric methods for indirect analysis and quantification of gametogenesis in *Chlamydomonas reinhardtii* (*Chlorophyceae*). PloS One 11, e0161453. doi: 10.1371/journal.pone.0161453 27676075 PMC5038954

[B59] Singh R. D. Sethy S. Ghosh S. Srivastava A. K. (2022). UV And γ-radiation induced molecular changes for rapid lipid accumulation in *Chlorella sorokiniana* . Biomass Bioenerg. 163, 106493. doi: 10.1016/j.biombioe.2022.106493

[B60] Song B. S. Kim B. K. Yoon Y. M. Jung K. Park J. H. Kim J. K.. (2016). Identification of red pepper powder irradiated with different types of radiation using luminescence methods: A comparative study. Food Chem. 200, 293–300. doi: 10.1016/j.foodchem.2016.01.050 26830591

[B61] Tang F. R. Loke W. K. Khoo B. C. (2017). Low-dose or low-dose-rate ionizing radiation-induced bioeffects in animal models. J. Radiat. Res. 58, 165–182. doi: 10.1093/jrr/rrw120 28077626 PMC5439383

[B62] Tran Q. G. Cho K. Park S. B. Kim U. Lee Y. J. Kim H. S. (2019). Impairment of starch biosynthesis results in elevated oxidative stress and autophagy activity in *Chlamydomonas reinhardtii* . Sci. Rep. 9, 9856. doi: 10.1038/s41598-019-46313-6 31285472 PMC6614365

[B63] Vanhoudt N. Horemans N. Wannijn J. Nauts R. Van Hees M. Vandenhove H. (2014). Primary stress responses in *Arabidopsis thaliana* exposed to gamma radiation. J. Environ. Radioact. 129, 1–6. doi: 10.1016/j.jenvrad.2013.11.011 24333636

[B64] Villanueva R. Chen Z. J. (2019). ggplot2: Elegant graphics for data analysis (2nd ed.). Measurement: Interdiscip. Res. Perspect. 17, 160–167. doi: 10.1080/15366367.2019.1565254

[B65] Vishwakarma J. Vavilala S. L. (2019). Evaluating the antibacterial and antibiofilm potential of sulphated polysaccharides extracted from green algae *Chlamydomonas reinhardtii* . J. Appl. .Microbiol. 127, 1004–1017. doi: 10.1111/jam.14364 31260145

[B66] Vítová M. Bišová K. Umysová D. Hlavová M. Kawano S. Zachleder V.. (2011). *Chlamydomonas reinhardtii*: duration of its cell cycle and phases at growth rates affected by light intensity. Planta 233, 75–86. doi: 10.1007/s00425-010-1282-y 20922544

[B67] Wase N. Tu B. Rasineni G. K. Cerny R. Grove R. Adamec J.. (2019). Remodeling of *Chlamydomonas* metabolism using synthetic inducers results in lipid storage during growth. Plant Physiol. 181, 1029–1049. doi: 10.1104/pp.19.00758 31501300 PMC6836844

[B68] Xiao Z. Storms R. Tsang A. (2006). A quantitative starch-iodine method for measuring alpha-amylase and glucoamylase activities. Anal. Biochem. 351, 146–148. doi: 10.1016/j.ab.2006.01.036 16500607

[B69] Yan N. Fan C. Chen Y. Hu Z. (2016). The potential for microalgae as bioreactors to produce pharmaceuticals. Int. J. Mol. Sci. 17, 962. doi: 10.3390/ijms17060962 27322258 PMC4926494

[B70] Yang L. Chen J. Qin S. Zeng M. Jiang Y. Hu L.. (2018). Growth and lipid accumulation by different nutrients in the microalga *Chlamydomonas reinhardtii* . Biotechnol. Biofuels 11, 40. doi: 10.1186/s13068-018-1041-z 29456627 PMC5809890

[B71] Zhang J. Muller B. S. F. Tyre K. N. Hersh H. L. Bai F. Hu Y.. (2020). Competitive growth assay of mutagenized *Chlamydomonas reinhardtii* compatible with the international space station veggie plant growth chamber. Front. Plant Sci. 11, 631. doi: 10.3389/fpls.2020.00631 32523594 PMC7261848

