# Supplementary material for: Application of ionizing radiation as an elicitor to enhance the growth and metabolic activities in Chlamydomonas reinhardtii
Source: Front Plant Sci. 2023 Feb 20;14:1087070. doi: 10.3389/fpls.2023.1087070 (PMC9986495; doi:10.3389/fpls.2023.1087070)
Supplement: Supplementary file 1 [file DataSheet_1.pdf]

Supplementary Material

1 Supplementary Figures and Tables

1.1 Supplementary Figures

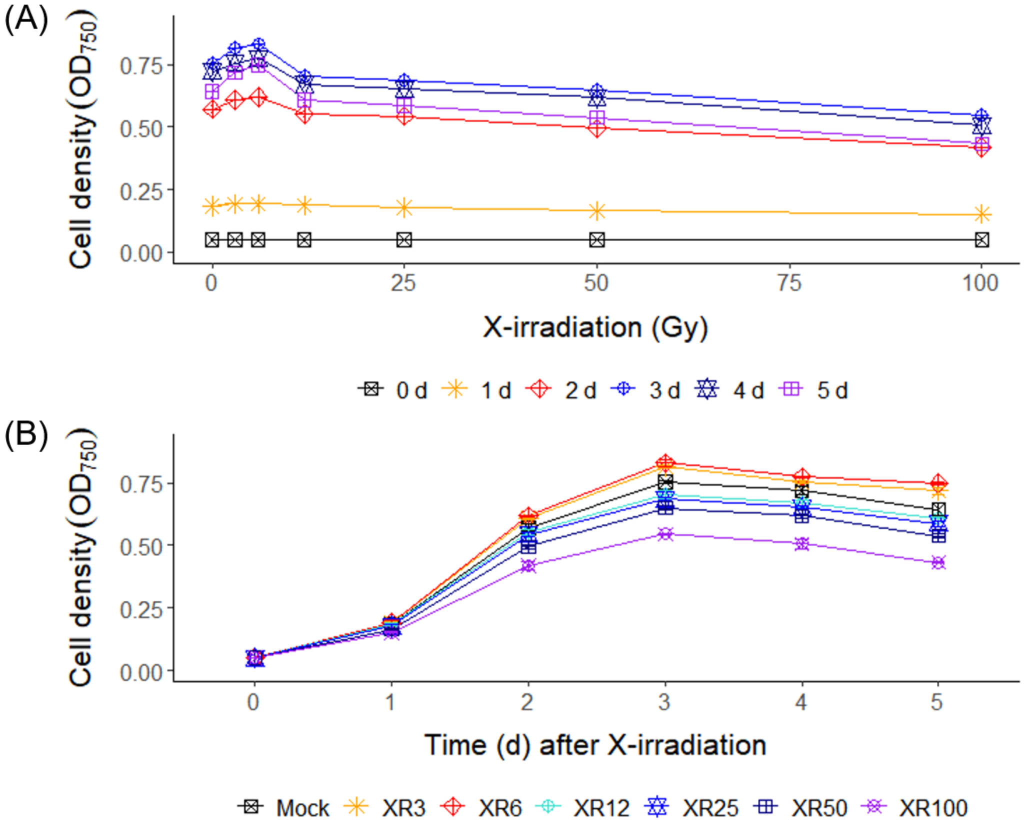

Supplementary Figure 1. Dose-dependent **(A)** or time-course growth **(B)** of *C. reinhardtii* cells after X-irradiation with different doses. Mid-exponential phase *C. reinhardtii* cells were differentially subjected to X-irradiation to have the absorbed doses of 3, 6, 12, 25, 50, and 100 Gy as described in the Materials and Methods section. XR3, XR6, XR12, XR25, XR50, and XR100 represent X-rays of 3, 6, 12, 25, 50, and 100 Gy, respectively. The cell densities were compared by measuring the optical density at 750 nm (OD_{750}) to evaluate the growth rates of mock and irradiated cells. Data represent the mean ± standard error (SE) with n = 9 from three independent experiments. Invisible error bars are smaller than the symbol size.

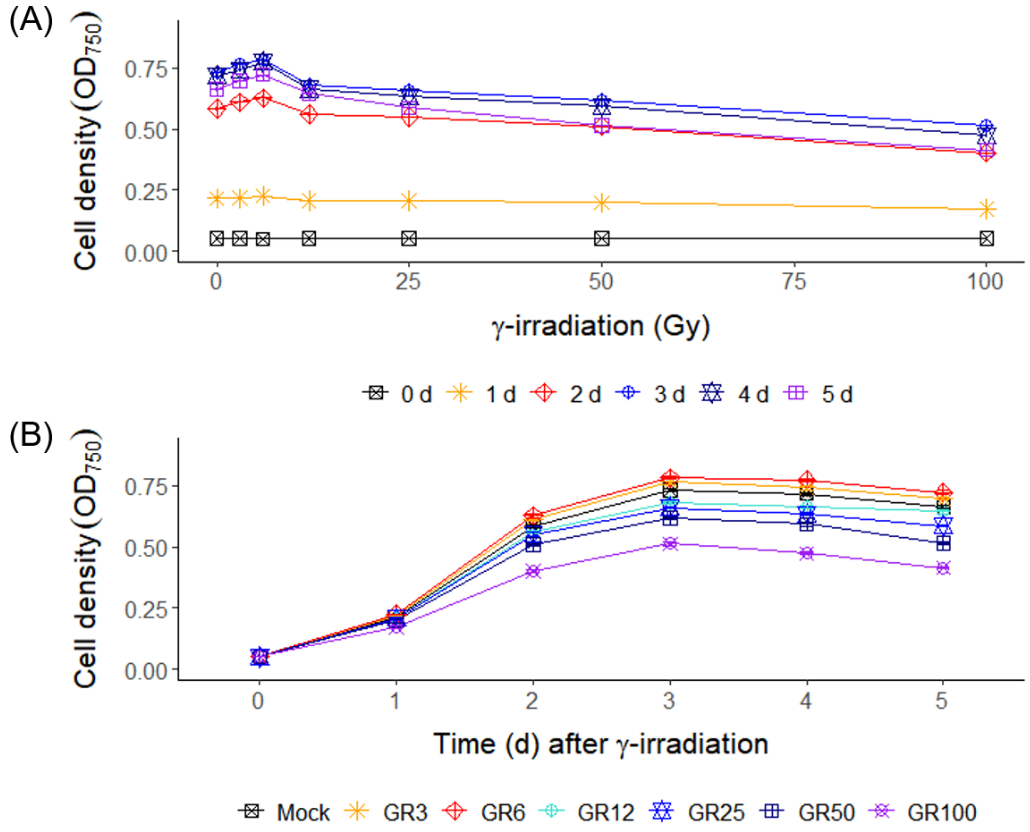

Supplementary Figure 2. Dose-dependent **(A)** or time-course growth **(B)** of *C. reinhardtii* cells after γ-irradiation with different doses. GR, γ-ray. Mid-exponential phase *C. reinhardtii* cells were differentially subjected to γ-irradiation to have the absorbed doses of 3, 6, 12, 50, and 100 Gy as described in the Materials and Methods section. GR3, GR6, GR12, GR25, GR50, and GR100 represent γ-rays of 3, 6, 12, 25, 50, and 100 Gy, respectively. The cell densities were compared by measuring the optical density at 750 nm (OD_{750}) to evaluate the growth rates of mock and irradiated cells. Data represent the mean ± standard error (SE) with n = 9 from three independent experiments. Invisible error bars are smaller than the symbol size.

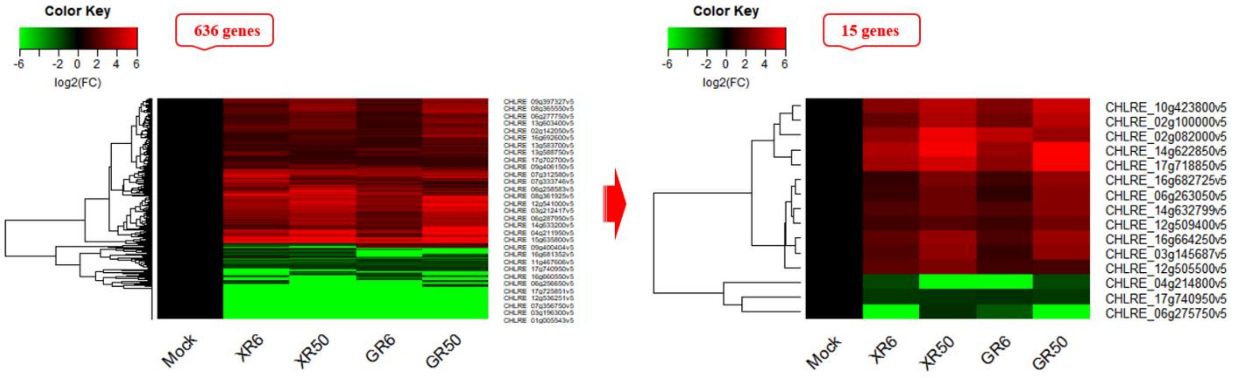

Supplementary Figure 3. Heat map analysis of genome-wide transcriptomic changes in *C. reinhardtii* cells after X- or γ-irradiation with two different doses. In total, 636 genes had a higher than two-fold transcription change in both X- and γ-irradiated groups, and, among them, only 15 had a descriptive annotation for gene name, as shown in **Table 1**. XR6 (GR6) and XR50 (GR50) represent X-rays (γ-rays) of 6 and 50 Gy, respectively. The color key indicates the log_2-transformation of normalized fold change values. The overall transcriptomic changes differed by the radiation dose rather than the radiation type.

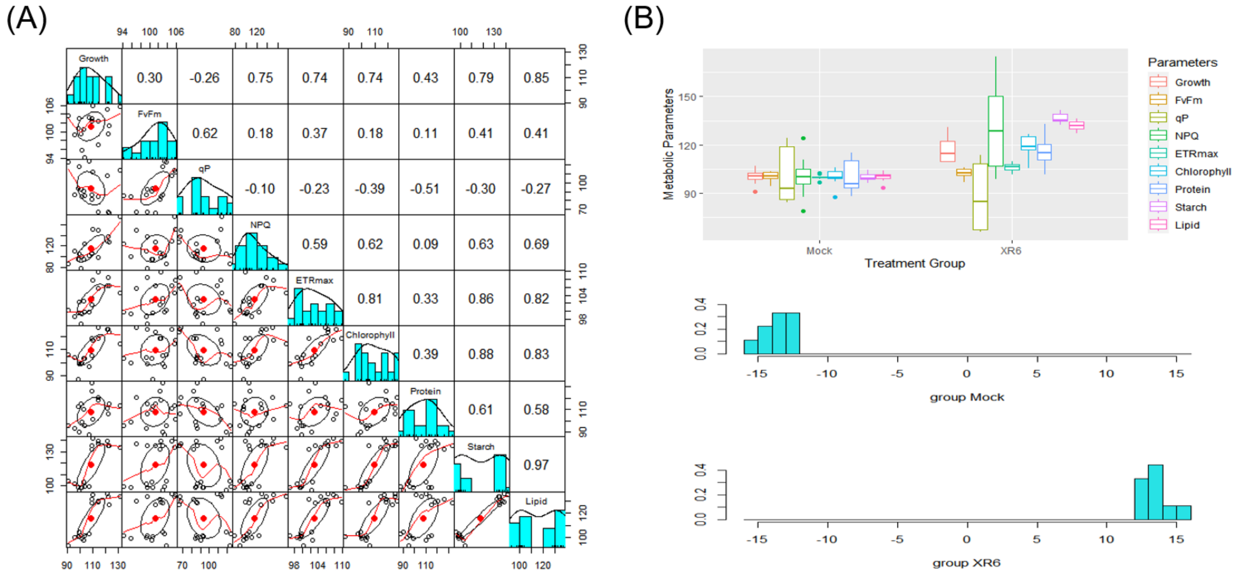

Supplementary Figure 4. Correlation **(A)** and discriminant **(B)** analyses of metabolic parameters including growth rate, photosynthetic activity, chlorophyll, protein, starch, and lipid contents between the mock and X-irradiated groups of *C. reinhardtii* cells. All measured values were normalized relative to the mock. The chlorophyll fluorescence and quenching parameters Fv/Fm, qP, NPQ, and the maximal electron transport rate ETRmax were used to evaluate photosynthetic activity. **(A)** Digits in grid are correlation values. **(B)** Stacked histograms represent discriminant function values. XR6, X-rays of 6 Gy. The data analysis and visualization were performed using the statistical and graphical functions of R 4.2.1 and ggplot2 in RStudio 2022.07.1+554, as described in the Materials and Methods section.

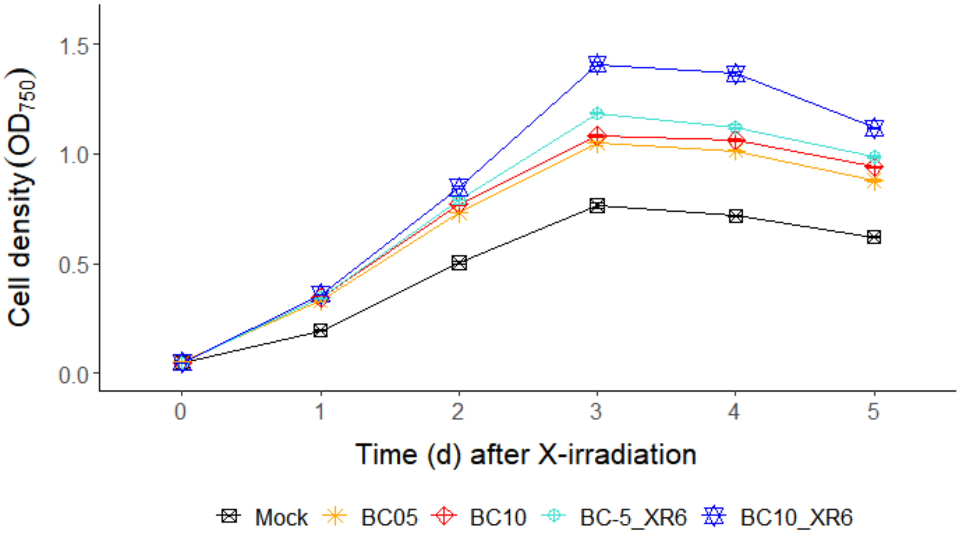

Supplementary Figure 5. Synergistic growth stimulation of *C. reinhardtii* cells in TAP medium containing sodium bicarbonate for 5 days after X-irradiation. BC05, BC10, and XR6 represent 5 and 10 mM $NaHCO_3$ and X-rays of 6 Gy, respectively. Data represent the mean ± standard error (SE) with $n = 3$ from three biological replicates. Invisible error bars are smaller than the symbol size.

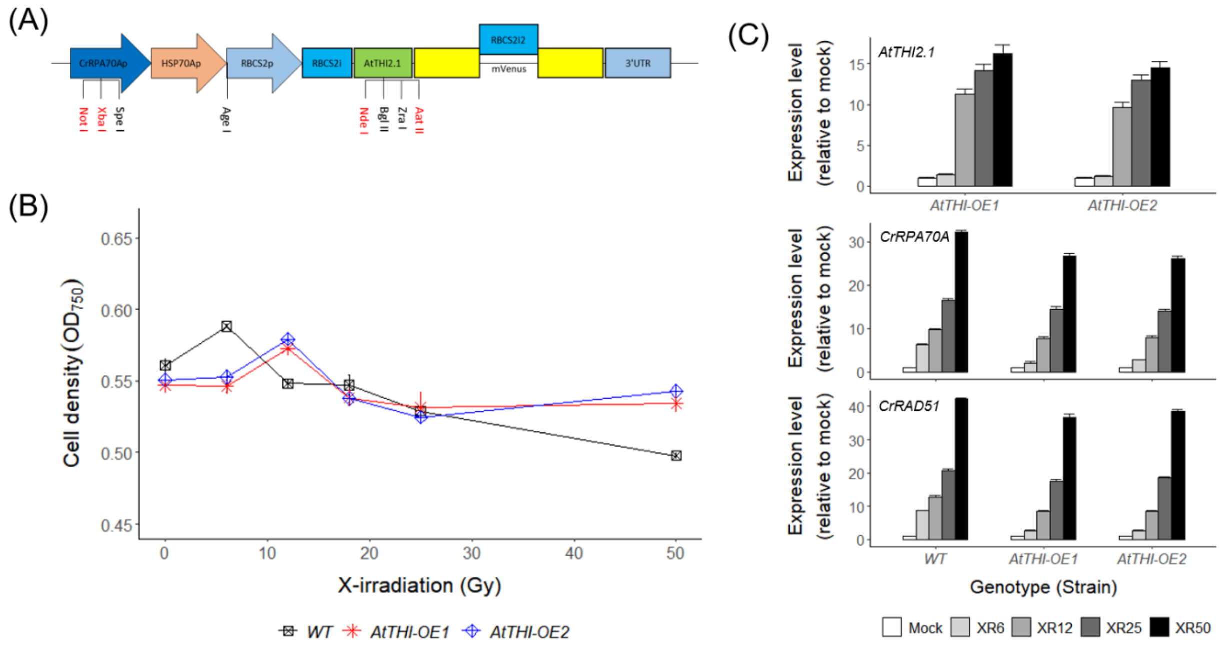

Supplementary Figure 6. Differences in cell growth of wild type and transgenic lines of *C. reinhardtii* in liquid TAP medium after X-irradiation with different doses. **(A)** Expression construct of CrRPA70Ap-HSP70Ap-RBCS2p-AtTHI2.1-pOpt2_mVenus to overexpress *AtTHI2.1* gene in *C. reinhardtii*. **(B)** Cell density was measured 2 days after X-irradiation with 6, 12, 18, 25, or 50 Gy. WT, wild type. **(C)** Relative transcription levels of a transgene *AtTHI2.1* and two DNA damage response genes *CrRPA70A* and *CrRAD51* after X-irradiation with 6, 12, 25, or 50 Gy. WT, wild type. XR6, XR12, XR25, and XR50 represent X-rays of 6, 12, 25, and 50 Gy, respectively. Data represent the mean ± standard error (SE) with *n* = 3 from three biological replicates. Invisible error bars are smaller than the symbol size.

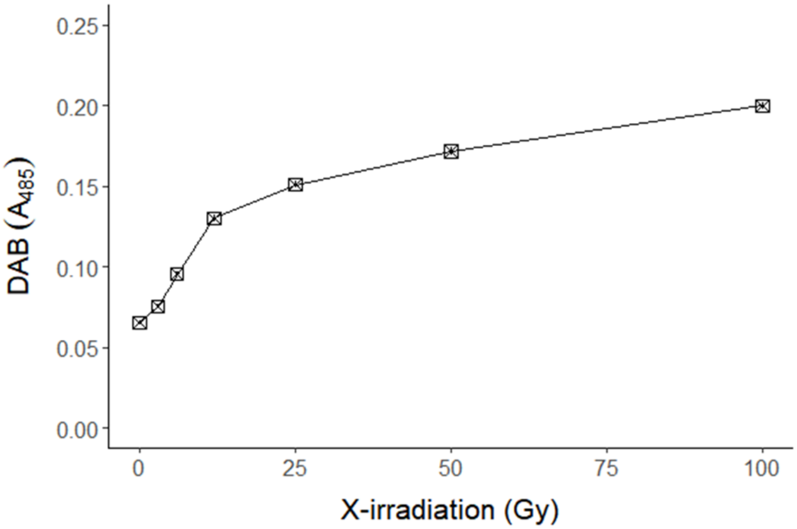

Supplementary Figure 7. Dose-dependent accumulation of H_2O_2 in TAP medium after X-irradiation with different doses and dose rates. TAP medium containing 5 mM 3,3'-diaminobenzidine (DAB), a radiation-stable semi-quantitative probe for H_2O_2, was subjected to X-irradiation of 3-12 or 25-100 Gy at the dose rate of 16.22 or 370.37 Gy h^{-1}, respectively. Since DAB forms deep brown polymerization products upon reaction with H_2O_2, the content of H_2O_2 in TAP medium was expressed by the absorbance at 485 nm (A_{485}) for polymerized DAB immediately after X-irradiation. Data represent the mean ± standard error (SE) with n = 6 from two independent experiments. Invisible error bars are smaller than the symbol size.

1.2 Supplementary Tables

Supplementary Table 1. Primer sequences used for RT-qPCR. Parentheses represent open reading frame (ORF) names, which are used to alphabetically list names temporarily attributed to an ORF by a sequencing project. *CrTUBA1* was used as an endogenous reference gene.

Gene (ORF) name	Primer sequence (forward / reverse)
CrRPA30 (CHLRE_02g100000v5)	5′-GCGGCGACATCCACTCGGACCT-3′ / 5′-CTGCATGACCTGCTGCAGGTTGT-3′
CrFEN1 (CHLRE_03g145687v5)	5′-CGGTCGTAAGGTTGCAGTAG-3′ / 5′-CATCAAAGACGTAGACGGGC-3′
CrKU (CHLRE_10g423800v5)	5′-TCGTTGGAACCAGTTTCCGT-3′ / 5′-TTAAAAGTGCACCTCGCCCA-3′
CrRAD51 (CHLRE_14g622850v5)	5′-GCCTGGTGGTTGTGGACAG-3′ / 5′-GTTGGCCACCACCTGATTG-3′
CrOASTL2 (CHLRE_16g664250v5)	5′-CGCTTGCCTTCGTCTCCTAA-3′ / 5′-CGGTACCACCTGGCATCAAA-3′
CrGST2 (CHLRE_16g682725v5)	5′-CAAGCTGACGGGTCTGTACC-3′ / 5′-AGCTGCTTCAGTTTGTCGCCG-3′
CrRPA70A (CHLRE_17g718850v5)	5′-GCACGACTTCAACGGCAG-3′ / 5′-GGTCAGGGACTGCTTGGC-3′
CrTUBA1 (CHLRE_03g190950v5)	5′-CTCGCTTCGCTTTGACGGTG-3′ / 5′-CGTGGTACGCCTTCTCGGC-3′
AtTHI2.1	5′-TACATGCCCTCGAGGTTGGGT-3′ / 5′-TCTGTACCAAGAGCTATGTAGTCCCAC-3′
